# First Report on some N_2_O_2_‐Donor Sets Tetradentate Schiff Base and Its Metal Complexes: Characterization and Antimicrobial Investigation

**DOI:** 10.1002/cbdv.202501117

**Published:** 2025-09-22

**Authors:** Amira A. Mohamed, Mohammed S. El‐Gedamy, Sadeek A. Sadeek, Hazem S. Elshafie

**Affiliations:** ^1^ Department of Basic Science Zagazig Higher Institute of Engineering and Technology Zagazig Egypt; ^2^ Department of Clinical Biochemistry and Molecular biology Urology and Nephrology Center Mansoura Egypt; ^3^ Department of Medical Laboratories Technology, College of Health and Medical Technologies Al‐Ayen Iraqi University (AUIQ), An Nasiriyah Thi‐Qar Iraq; ^4^ Department of Chemistry, Faculty of Science Zagazig University Zagazig Egypt; ^5^ Department of Agricultural, Forestry, Food and Environmental Sciences University of Basilicata Potenza Italy

**Keywords:** antimicrobial investigation, mass spectrometry (GCMS), optical band gap energy, synthesis of new Schiff base (H_2_L), UV–visible

## Abstract

The new Schiff base ligand *N*,*N*′‐thiophene‐(bis‐1‐ethyl‐6,8‐difluoro‐7‐(3‐methylpiperazin‐1‐yl)‐4‐oxo‐1,4‐dihydroquinoline‐3‐carboxylic acid) (H_2_L) was synthesized via condensation of thiophene‐2,5‐diamine with lomefloxacin in ethanol using glacial acetic acid. H_2_L reacted as tetradentate ligand with chromium(III), manganese(II), and cobalt(II). The synthesized compounds were characterized using elemental analysis, molar conductivity (Λ_m_), magnetic susceptibility (*µ*_eff_), FTIR, UV–Vis, GC–MS, and thermal studies were employed to ensure the chelation process. Infrared measurements confirmed that H_2_L chelated with metal ions via the carboxylate oxygen and nitrogen of the azomethine group also, with water molecules. UV–Vis and magnetic moment data supported octahedral geometries for all complexes. The calculated optical band gap energy (*E*_g_) values suggested that our complexes were more electro‐conductive relative to H_2_L. Thermal analyses revealed the presence of significant lattice and coordinated water molecules within the complexes' coordination spheres. Thermodynamic parameters including activation energy, entropy change, enthalpy change, and Gibbs free energy change were derived using Coats–Redfern and Horowitz–Metzger methods. Disc diffusion method was implemented for assessing antimicrobial effects versus some pathogenic microorganisms. The data revealed that Cr(III) complex as most effective against *Escherichia coli* and *Staphylococcus aureus*. Cytotoxicity tests on normal prostate epithelial cells (IC_50_ = 6.30–10.43 µM) indicated significant toxicity and limited selectivity, restricting anticancer applicability.

## Graphical abstract

Mass spectrometry, UV‐Vis. spectra, thermal analysis and antimicrobial evaluation were done for a novel tetradentate Schiff base ligand (H_2_L) and its metal complexes.

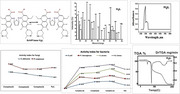

## 1 Introduction

**SCHEME 1:**
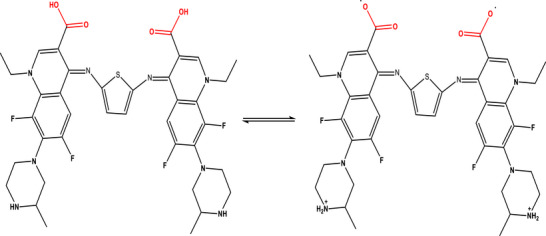
*N*,*N*′‐Thiophene (bis‐1‐ethyl‐6,8‐difluoro‐7‐(3‐methylpiperazin‐1‐yl)‐4‐oxo‐1,4‐dihydroquinoline‐3‐carboxylic acid) (H_2_L).

Schiff bases have long attracted scientific interest due to their versatile pharmacological and biological activities, primarily linked to their strong chelating ability and the presence of the azomethine (─CH═N─) functional group, which is critical for their bioactivity [1, 2, 3, 4, 5, 6]. Numerous studies were demonstrated that Schiff bases possess a wide range of therapeutic effects, including antibacterial, antifungal, antioxidant, anti‐inflammatory, anticancer, herbicidal, and anthelmintic properties, making them valuable candidates for drug development [7, 8]. Beyond their biological roles, Schiff bases were utilized in industrial applications such as catalysts, dyes, pigments, and polymer stabilizers [9, 10]. Structurally, Schiff bases were metabolized as bidentate, tridentate, tetradentate, or polydentate ligands with a wide variety of metal ions in different oxidation states, leading to enhanced chemical stability and biological activity [11, 12, 13, 14, 15]. In the pharmaceutical field, the Schiff base metal complexes were demonstrated enhanced pharmacological performance, including antibacterial, antifungal, anticancer, antiviral, anticonvulsant, and anti‐HIV activities [7, 16, 17, 18, 19, 20, 21, 22, 23, 24, 25, 26, 27]. Recent research for Schiff base metal complexes were highlighted significance in different area such as DNA interaction studies, chemotherapeutics, supramolecular chemistry, pharmaceutical development, material science, and biomedical technologies [27, 28]. Fluoroquinolones were emerged as a significant class of synthetic antibacterial drugs [29, 30, 31, 32, 33]. Also, fluoroquinolones were effective against a wide range of harmful pathogens and are resistant to tetracyclines, aminoglycosides, penicillins, cephalosporins, and other antibiotics [33, 34, 35]. Lomefloxacin, is one of the third‐generation fluoroquinolone, has significant efficacy versus an extensive variety of negative and Gram‐positive pathogens [34, 35]. A survey of the scientific literature discloses that no prior study has reported for the synthesis or biological evaluation of Schiff bases derived from lomefloxacin and thiophene‐2,5‐diamine, so the novelty for our work is building on synthetic new Schiff base *N*,*N*′‐thiophene (bis‐1‐ethyl‐6,8‐difluoro‐7‐(3‐methylpiperazin‐1‐yl)‐4‐oxo‐1,4‐dihydroquinoline‐3‐carboxylic acid) (H_2_L) (Scheme 1), including its complexes with chromium(III), manganese(II), and cobalt(II). The mechanism of bonding of H_2_L with Cr(III), Mn(II), and Co(II) was investigated. Several spectroscopic methods were used to perform the characterization. The techniques used for characterization including FTIR, UV–Vis, elemental analysis, magnetic moments, conductance studies, mass spectroscopy, and thermal analyses. The antimicrobial and antifungal activities of these compounds were investigated against selected pathogenic strains, including *Bacillus cereus*, *Staphylococcus aureus*, *Escherichia coli*, *Pseudomonas aeruginosa*, *Candida albicans*, and *Penicillium vulpinum*, using the standardized disc diffusion method. To explore the potential anticancer relevance and safety profile of the synthesized compounds, cytotoxicity was assessed in vitro against normal human prostate epithelial cells (PrEC) employing the 3‐(4,5‐dimethylthiazol‐2‐yl)‐2,5‐diphenyl tetrazolium bromide (MTT) assay. This approach aimed to determine the degree of cellular toxicity and evaluate whether the compounds exhibit any degree of selectivity, which is essential for their future development as therapeutic agents with antimicrobial or anticancer applications.

## 2 Materials and Methods

### 2.1 Materials

The chemicals handled were certified as analytical reagents (AR) and of the highest possible accuracy obtainable. Lomefloxacin (LFX), thiophene 2‐5 diamine (thio‐en), chromium chloride dihydrate, manganese chloride monohydrate and cobalt chloride hexahydrate, potassium dichromate, silver nitrate, absolute ethyl alcohol, dimethyl sulfoxide (DMSO‐d_6_), and dimethylformamide (DMF) they supplied from Obour Pharmaceutical Industrial, Sigma, and Aldrich Chemicals, correspondingly. Glasses were immersed in a chromic combination (potassium dichromate + concentrated sulfuric acid) during the night and then thoroughly cleaned with bidistilled water and dry in an oven at 100°C.

### 2.2 Synthesis of *N*,*N*′‐Thiophene (bis‐1‐ethyl‐6,8‐difluoro‐7‐(3‐methylpiperazin‐1‐yl)‐4‐oxo‐1,4‐dihydroquinoline‐3‐carboxylic acid Schiff Base (H_2_L)

**SCHEME 2:**
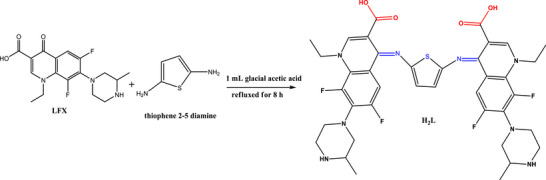
Synthesis of H_2_L Schiff base.

The pale yellow Schiff base ligand (H_2_L) was synthesized through a condensation reaction between LFX and thio‐en in ethanol. LFX (2 mmol, 1.562 g) and thio‐en (1 mmol, 0.114 g) were dissolved in 50 mL of ethanolic solution with glacial acetic acid as catalyst (Scheme 2). The reaction mixture was then refluxed under continuous stirring for 8 h. Upon completion, the mixture was cooled in an ice‐water bath to 0°C. The pale yellow solid product was isolated by vacuum filtration, washed with ethanol and dried under vacuum over anhydrous calcium chloride.

### 2.3 Synthesis of Metal Complexes

The dark green [Cr(H_2_L)(H_2_O)_2_]Cl_3_·4H_2_O, (**1**) was prepared by mixing 0.5 mmol (0.3890 g) of H_2_L in 30 mL absolute ethanol with 0.5 mmol (0.1589 g) of CrCl_3_·2H_2_O in 20 mL ethanol. The mixture was refluxed for 10 h, and the precipitate was filtered out and dried under vacuum on anhydrous calcium chloride. The pale yellow and pale green solid complexes [Mn(H_2_L)(H_2_O)_2_]Cl_2_·4H_2_O (**2**) and [Co(H_2_L)(H_2_O)_2_]Cl_2_·5H_2_O (**3**) were made in the exact same way mentioned above, employing MnCl_2_·H_2_O and CoCl_2_·6H_2_O.

### 2.4 Instruments

A PerkinElmer 2400 CHN elemental analyzer was used to carry out the analyses. Vogel's textbook described methods were used to determine metal and chloride contents [36]. Also, the fraction of metal ions was estimated gravity measurement through transformation of the solid products into metal oxide and using an atomic absorption approach [29, 30]. For this aim, a spectrometer type PYE‐UNICAM SP 1900 equipped with the matching lamp was employed. FTIR spectra in KBr discs were acquired in the 4000–400 cm^−1^ utilizing an FTIR 460 PLUS Spectrophotometer. TG‐DTG investigations were conducted beneath N_2_ atmosphere at temperatures ranging from room temperature to 1000°C employing a Shimadzu TGA‐50H. The sample mass was adequately weighed in an aluminum crucible. The UV‐3101PC Shimadzu electronic spectrometer was used. The absorption spectra were captured as solutions in DMSO‐d_6_. The magnetic susceptibilities of the powdered materials were measured at room temperature using a Sherwood scientific magnetic balance and a Gouy balance with Hg[Co(CSN)_4_] as the calibrant. Mass spectra were acquired utilizing a Shimadzu GCMS‐QP‐1000EX (ESI‐70ev) over the range of 0–1090. Melting points were captured using a Buchi apparatus. The molar conductance of 1 × 10^−3^ M solutions of H_2_L and their complexes in DMF was assessed at room temperature utilizing CONSORT K410. Every measurement was performed at room temperature, assuming newly created samples.

### 2.5 Antimicrobial Investigation

#### 2.5.1 Antibacterial Activity Assay

Gram‐positive bacterial strains *S. aureus* (ATCC 6538) and *B. cereus* (GST4), as well as Gram‐negative strains *E. coli* and *P. aeruginosa*, were employed in the evaluation of the antimicrobial efficacy of the synthesized Schiff base ligand (H_2_L) and its corresponding metal complexes. The bacterial strains were obtained and maintained at the Department of Agricultural, Forestry, Food, and Environmental Sciences (DAFE), University of Basilicata, Potenza, Italy. Antimicrobial activity was assessed using a modified disc diffusion method described by Beecher and Wong [37]. Müller–Hinton agar served as the nutrient medium [38, 39]. The agar medium was sterilized, cooled to 47°C, and then inoculated with the respective bacterial cultures. To ensure a uniform diffusion of the compounds, a thin sterile agar overlay was poured over the primary inoculated agar layer in 12 cm Petri dishes containing 15 mL of medium. Sterile 5 mm diameter filter paper discs impregnated with the test compounds (prepared at a concentration of 1.0 × 10^−3^ M in DMF; 0.1 mL per disc) were carefully placed on the surface of the solidified agar. The plates were incubated at 37°C for 20 h. Antibacterial efficacy was quantified by measuring the diameters of the inhibition zones (in mm). Ciprofloxacin and amikacin were used as positive control antibiotics to validate bacterial susceptibility. The percentage activity index of each compound was calculated relative to the inhibition zone produced by the standard antibiotics using Equation (1):

(1) $$\begin{array}{ccc} & & \%\;\mathrm{Activity}\;\mathrm{index}\\ & & \;=\frac{\mathrm{Zone}\;\mathrm{of}\;\mathrm{inhibition}\;\mathrm{by}\;\mathrm{test}\;\mathrm{compound}\;\left(\mathrm{diameter}\right)}{\mathrm{Zone}\;\mathrm{of}\;\mathrm{inhibition}\;\mathrm{by}\;\mathrm{standard}\;\left(\mathrm{diameter}\right)}\times 100 \end{array}$$

#### 2.5.2 Antifungal Activity Assay

The antifungal efficacy of the synthesized H_2_L and its metal complexes was evaluated against two fungal species: *C. albicans* (OC10) and *P. vulpinum* (CM1). Pure cultures of both fungi were maintained at 4°C on Potato Dextrose Agar (PDA) and preserved in the mycotheca of DAFE [40, 41]. Identification of the fungal strains was confirmed using both morphological characteristics and molecular diagnostic methods, consistent with established taxonomic protocols. The antifungal assay was performed using the disc diffusion method, as described in previous studies [38, 39]. PDA plates were inoculated with standardized fungal spore suspensions, followed by the placement of sterile filter paper discs (5 mm diameter) impregnated with the test compounds (1.0 × 10^−3^ M in DMF; 0.1 mL per disc). The plates were incubated at 28°C for 48 h under controlled conditions to allow for fungal growth and compound diffusion. Antifungal activity was determined by measuring the diameter of the inhibition zones (in mm) surrounding the discs. Commercial antifungal agent clotrimazole was used as a positive reference standard to assess the relative sensitivity of the tested fungal pathogens. The activity index (%) of each compound was calculated in comparison to the inhibition zone produced by clotrimazole, providing a quantitative measure of antifungal potency.

### 2.6 Cytotoxicity Assay

#### 2.6.1 Cell Culture

To evaluate the cytotoxicity of H_2_L and its metal complexes on normal human cells, preliminary assays were conducted using a normal prostate epithelial cell line (PrEC). The PrEC cell line was obtained from the Egyptian VACSERA Inc. and cultured in Eagle's Minimum Essential Medium (EMEM; Bio‐Whittaker‐Lonza, Switzerland), supplemented with 10% (v/v) fetal calf serum (FCS) and 1% penicillin–streptomycin solution (100 IU/mL). Cells were maintained under standard culture conditions at 37°C in a humidified atmosphere containing 5% CO_2_ and 95% air. Subculturing was routinely performed to maintain logarithmic growth before cytotoxicity experiments. Cell line handling procedures were carried out in compliance with the guidelines of the Institutional Review Board (IRB), Faculty of Veterinary Medicine, Zagazig University, Egypt.

#### 2.6.2 MTT Assay

The cytotoxic potential of H_2_L and its metal complexes was assessed using the MTT colorimetric assay. PrEC cells were seeded in 96‐well plates at a density of 5 × 10^4^ cells/mL (100 µL per well) and allowed to adhere overnight. Following initial incubation, cells were exposed to serial dilutions (12.5, 25, 50, 100, and 200 µM) of the test compounds for 48 h under the same incubation conditions. After treatment, the culture medium was replaced with 100 µL of fresh medium containing MTT solution (0.5 mg/mL), and the plates were incubated for an additional 4 h. Viable cells reduced MTT to insoluble purple formazan crystals, which were subsequently solubilized by the addition of 150 µL of 10% sodium dodecyl sulfate (SDS) in 0.01 N HCl. The absorbance of each well was measured at 570 nm using a Biotek ELISA plate reader (Gen5, USA).

Cell viability was calculated using the Equation (2):

(2) $$\%\;\mathrm{Cell}\;\mathrm{viability}=\frac{\mathrm{Absorbance}\;\mathrm{of}\;\mathrm{treated}\;\;\mathrm{cells}\;}{\mathrm{Absorbance}\;\mathrm{of}\;\mathrm{control}\;\mathrm{cells}\;}\times 100$$

The half‐maximal inhibitory concentration (IC_50_), defined as the concentration of compound required to inhibit 50% of cell viability, was determined for each tested compound.

## 3 Results and Discussion

The synthesized metal complexes of Cr(III), Mn(II), and Co(II) with a high yield (76.85%–89.57%) were stable at room temperature, soluble in DMF, DMSO‐d_6_ and have high melting points from 279°C to 290°C. Table 1 summarizes physical and analytical data for H_2_L and its metal complexes. Molecular formulae acquired through microanalysis, mass studies, and thermal analyses (TG/DTG). The data confirmed that all synthesized complexes exhibit a 1:1 molar ratio of ligand to metal ion and contain coordinated and/or lattice water molecules. The molar conductivity of H_2_L was found at 10.23 Ω^−1^ mol^−1^ cm^2^, which is consistent with its non‐electrolytic nature. In contrast, the metal complexes displayed significantly higher conductance values, ranging from 180.36 to 264.25 Ω^−1^ mol^−1^ cm^2^. These values are indicative that Complex (**1**) behaves as a 1:3 electrolyte, while both Complexes (**2**) and (**3**) found as 1:2 electrolytes, in agreement with established literature values for similar coordination compounds [5, 18, 42]. Also, the presence of chloride outside the sphere was confirmed by qualitative reaction. Solution of silver nitrate reacts with solution of their complexes giving a white precipitate of silver chloride which in good agreement with the molar conductivity measurements.

**TABLE 1:** Physico‐analytical data for H_2_L and its metal complexes.

Compounds	M.Wt. (M.F.)	Yield%	Mp (°C)	Color	Found (Calcd.) %					Λ_m_ (Ω^−1^ mol^−1^ cm^2^)
					C	H	N	M	Cl	
H_2_L	778.00 (C_38_H_38_F_4_N_8_O_4_S)	81.24	265	Pale yellow	58.39 (58.61)	5.09 (4.88)	14.29 (14.40)	—	—	10.23
(**1**) [Cr(H_2_L)(H_2_O)_2_]Cl_3_·4H_2_O	1044.34 (CrC_38_H_50_F_4_N_8_O_10_SCl_3_)	85.74	285	Dark green	43.50 (43.66)	4.91 (4.79)	11.64 (10.72)	4.92 (4.98)	10.08 (10.18)	264.25
(**2**) [Mn(H_2_L)(H_2_O)_2_]Cl_2_·4H_2_O	1011.83 (MnC_38_H_50_F_4_N_8_O_10_SCl_2_)	79.80	279	Pale yellow	44.92 (45.07)	5.08 (4.94)	10.99 (11.07)	5.38 (5.43)	6.92 (7.00)	180.36
(**3**) [Co(H_2_L)(H_2_O)_2_]Cl_2_·5H_2_O	1033.83 (CoC_38_H_52_F_4_N_8_O_11_SCl_2_)	84.36	290	Pale green	43.93 (44.11)	5.14 (5.03)	10.76 (10.83)	5.62 (5.03)	6.79 (6.86)	182.76

The chloride content in the complexes was determined by using two methods. *Mohr's method*: The complexes solutions were estimated volumetrically according to the Mohr method which is based on titration of chloride with standard solution of AgNO_3_ in the presence of K_2_CrO_4_ as indicator. *Volhard's method*: The complexes solutions were estimated volumetrically according to back titration, which involves an additional excess of silver nitrate to the complex solution; this excess silver nitrate is titrated against ammonium thiocyanate, with ferric ammonium sulfate as an indicator.

### 3.1 FTIR Spectral Analysis and Coordination Mode

**SCHEME 3:**
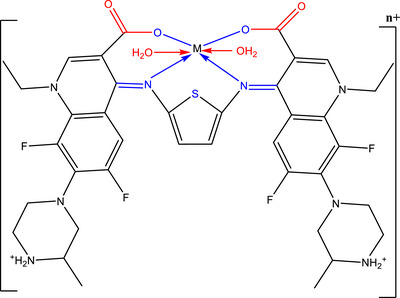
Coordination mode of Cr(III), Mn(II), and Co(II) with H_2_L. *n* = 3 for Cr(III) and 2 for Mn(II), and Co(II).

Infrared spectra (Figure S1) were employed to elucidate the coordination behavior of (H_2_L) ligand with Cr(III), Mn(II), and Co(II) ions. The most significant spectra of free H_2_L and its corresponding metal complexes were summarized in Table 2. The Infrared spectrum of H_2_L was thoroughly investigated to assess the efficacy of metal ion binding at different vibration frequencies. A broad absorption band observed between 3353 and 3500 cm^−1^ can be ascribed to O─H stretching vibrations of both coordinated water molecules and the carboxylic acid of H_2_L [5, 18, 21, 43]. Furthermore, the inclusion of bands around 845 and 608 cm^−1^ which associated to the rocking and wagging vibration of coordinated water supporting all the presence of water in all complexes [21, 39, 40, 41, 42, 43, 44, 45]. The *ν*(N─H) vibration of ^+^NH_2_ quaternized nitrogen in the piperazinyl group at 2939–2460 cm^−1^ indicating that the zwitterionic nature of H_2_L in its coordinated form [34, 35]. The IR spectra of H_2_L reveals two bands in 1721 and 1630 cm^−1^, corresponding to the stretching vibrations of the carboxylic *ν*(COOH) and azomethine group *ν*(─CH═N─) [21, 44, 46, 47]. A absence of the band at 1721 cm^−1^ in all complexes, and the blue shift for the azomethine group from 1630 to 1524 cm^−1^ indicate that one oxygen atom of the carboxylate group and nitrogen atom of the azomethine group *ν*(─CH═N─) in coordination with the metal ions to forming six‐membered rings (Scheme 3) [21, 44, 48, 49, 50]. The occurrence of stretching vibrations *ν*_as_(COO^−^) in the 1612–1603 cm^−1^ zone and *ν*_s_(COO^−^) in the 1404–1390 cm^−1^ ensemble with Δ*ν* > 200 cm^−1^ demonstrates that the carboxylate group link as monodentate across one of the oxygen atoms in our complexes [21, 44, 51, 52]. At the range of 763–512 cm^−1^, new bands emerged in the spectra of complexes, which were associated with *ν*(M─O) and *ν*(M─N) stretching vibrations, confirming the coordination of both azomethine nitrogen and carboxylate oxygen to the metal centers [53].

**TABLE 2:** Selected infrared wavenumbers (cm^−1^) for H_2_L and its metal complexes.

Compounds	*ν*(O─H); H_2_O; COOH	*ν*(C═O); COOH	*ν*_as_(COO^−^)	*ν*(C═N)	*ν*_s_(COO^−^)	*ν*(M─O) and *ν*(M─N)
H_2_L	3500m	1721vs	—	1630s	—	
(1)	3353m	—	1612s	1535w	1404w	763m and 512w
(2)	3401mbr	—	1610vs	1524s	1391m	738m and 512w
(3)	3420mbr	—	1603vs	1528s	1390m	690m and 513w

*Note*: Keys: s = strong, w = weak, m = medium, br = broad, *ν* = stretching.

### 3.2 UV–Visible Absorption Spectroscopy and Magnetic Susceptibility Studies

UV–Vis spectra of free H_2_L and its metal complexes (Figure S2) were acquired in dimethyl sulfoxide within 200–800 nm for better comprehension of the electronic structure of our compounds. The transitions (π–π* and *n*–π*) for H_2_L were found at 34482 and 31746 cm^−1^ (Table 3). These transitions happen within unsaturated hydrocarbons possessing ketone or azomethine groups [54, 55]. The complexes revealed distinctive bands between 22 222 and 20 408 cm^−1^, presumably resulting from ligand–metal charge transfer (MLCT) [56]. UV–Vis spectrum of Cr(III) complex shows two absorption bands at 18 867 and 17 211 cm^−1^, depicting the ^6^A_2g_→^4^T_2g_ (F) and ^4^T_2g_→^4^T_1g_ (F) transitions. Correspondingly with apparent magnetic moment appreciates of 3.82 B.M. implies an octahedral geometry [54]. The electronic spectrum of Mn(II) complex revealed two identifiable bands at 19 417 and 16 528 cm^−1^, corresponding to ^6^A_1g_→^4^T_1g_ (4G) and ^4^A_1g_→^4^E_g_, ^4^A_1g_ (4G) transitions with *µ*_eff_ value at 1.80 B.M., suggesting the complex's low octahedral spin [55, 56]. Co(II) complex shows an identifiable band at 18 018 cm^−1^ which corresponds to the ^4^T_1g_ (F)→^4^T_1g_ (P) transition which is consistent with the anticipated band for six‐coordinate Co(II) complexes [54, 55, 56]. Molar absorptivity (*ε*) for complexes was estimated using the relation: *A* = *ɛCl*, where *A* is the absorbance, *C* = 1 × 10^−3^ M, and *l* is the length of cell (1 cm).

**TABLE 3:** UV–visible spectral data of H_2_L and its corresponding metal complexes.

Compounds	Peak		Assignment	ε* (M^−1^ cm^−1^) × 10^4^	10Dq		CFSE#	*µ*_eff_ (B.M.)
	nm	cm^−1^			cm^−1^	kJ/mol		
H_2_L	290	34 482	π→π*	451				
	315	31 746	*n*→π*	155				
(**1**)	296	33 783	π→π*	464	18 867	225	−270	3.82
	330	30 303	π→π*	160	17 211	206	−247	
	485	20 618	CT	50				
	530	18 867	^6^A_2g_→^4^T_2g_	60				
	581	17 211	^4^T_2g_→^4^T_1g_	80				
(**2**)	295	33 898	π→π*	452	19 417	232	−464 + 2p	1.80
	325	30 769	*n*→π*	158	16 528	198	−396 + 2p	
	456	21 929	CT	100				
	515	19 417	^6^A_1g_→^4^T_1g_	90				
	605	16 528	^4^A_1g_→^4^T_2g_	80				
(**3**)	294	34 013	π→π*	453	18 018	216	−172 + 2p	
	322	31 055	*n*→π*	170				
	490	20 408	CT	67				
	555	18 018	^4^T_1g_→^4^T_1g_	90				4.32

### 3.3 ^1^H NMR Spectral Analysis

The suggested molecular structures of the compounds were also proved by ^1^H NMR spectroscopy. In the present study, the ^1^H NMR spectra of H_2_L and its complexes were recorded in DMSO‐d_6_ (Figure S3) and the results were collected in Table 4. The assignment of every signal was achieved by comparing the ^1^H NMR spectra of the complexes to those of H_2_L. On comparing main peaks of H_2_L with its complexes, we observed that all the signals of the free H_2_L present in the ^1^H NMR spectra of all complexes except the signal at *δ* 11.22 ppm which corresponding to ─OH of the carboxylic group which disappeared in all complexes [3], the disappearance of this signal assigned to the chelation of H_2_L through carboxylic group [15, 18]. The triplet signals at *δ*: 1.12–1.31 ppm attributed to *δ*, ─CH_3_ aliphatic, singlet signal at *δ*: 1.91 ppm attributed to *δ*, ─NH, multiplet at *δ*: 7.17–8.86 ppm for ─CH aromatic and singlet signal at *δ*: 11.22 ppm corresponding to ─OH of carboxylate group respectively, the signal at *δ* 11.22 ppm disappeared in all complexes attributed to coordination of H_2_L to Cr(III), Mn(II), and Co(II) through the carboxylate group. Also, the ^1^H NMR spectra for complexes exhibit new signal in the range 4.33–4.45 ppm, due to the presence of water molecules in the complexes [51, 56].

**TABLE 4:** ^1^H NMR values (ppm) and tentative assignments for H_2_L and its metal complexes.

Assignments	H_2_L	(1)	(2)	(3)
*δ*, ─CH_3_ aliphatic	1.12–1.31	1.14–1.29	1.16–1.27	1.11–1.26
*δ*, ─NH; piperazine	1.91	1.90	1.92	1.94
*δ*, ─N─CH_2_	3.13–3.56	3.11–3.54	3.15–3.56	3.12–3.53
*δ*, H_2_O	—	4.36	4.33	4.45
*δ*, ─CH_2_ aromatic	7.17–8.86	7.15–8.69	7.10–8.99	7.14–8.68
*δ* ─OH, COOH	11.22	—	—	—

### 3.4 Optical Band Gap Energy

The optical band gap (*E*_g_) was determined according to Equation (3) [57]

(3) $$\alpha h\nu ={\left(h\nu -E_{g}\right)}^{n}$$

where *hν* is the photon energy, *h* is Planck's constant, *α* is the absorption coefficient, and *n* equals 1/2 or 2 for allowed direct and indirect electronic transitions.

**FIGURE 1:**
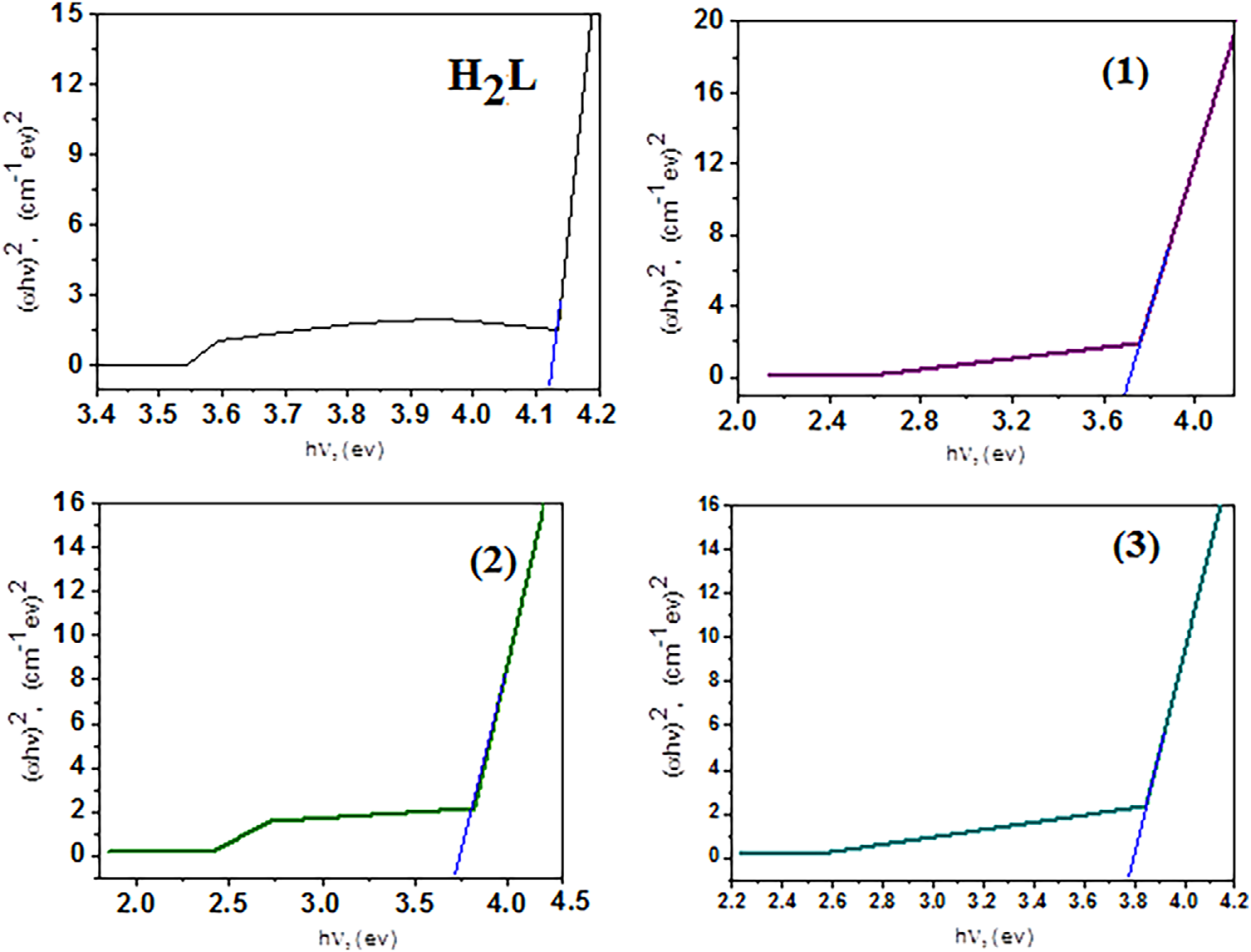
Allowed direct band gaps of H_2_L and its complexes.

Figure 1 demonstrates the plot of (*αhν*)^2^ against (*hν*) and *E*_g_ is derived by prolonging the linear section of the curve until (*αhν*)^1/2^ = 0. The (*α*) was calculated assuming the equation *α* = 1/*d*ln (1/*T*) (**4**), where *d* represents the cuvette's optical path length and *T* represented the estimated transmittance. In the DMF solvent, the *E*_g_ for H_2_L, Cr(III), Mn(II), and Co(II) compounds at first region and second region are 4.16, 3.62, 3.55, and 3.73 eV, respectively, and are mentioned in Figure 1. The data in Table 1 demonstrates that complexation reduces the *E*_g_ values of the complexes. The decrease in *E*_g_ is caused by electrons moving toward the metal [58]. It is proposed that Cr(III), Mn(II), and Co(II) increase the mobilization of ligand electrons by accepting them in their shell, thereby expanding the width of the localized levels in the resultant complex and reducing the *E*_g_. This finding has several applications in optics, electronics, and energy‐conversion devices [59]. In fact, a narrow *E*_g_ enhances the molecule's electroconductivity by enabling electronic transitions to occur between HOMO and LUMO energy levels [60]. According to the mentioned optical characteristics, the produced compounds may be employed as semiconductors which are found in the same range as highly efficient solar materials [61, 62]. Mn(II) complex with optical band gap (*E*_g_) 3.55 eV, the complex is suitable for optics, electronics, and energy‐conversion devices.

### 3.5 Mass Spectrometry

**SCHEME 4:**
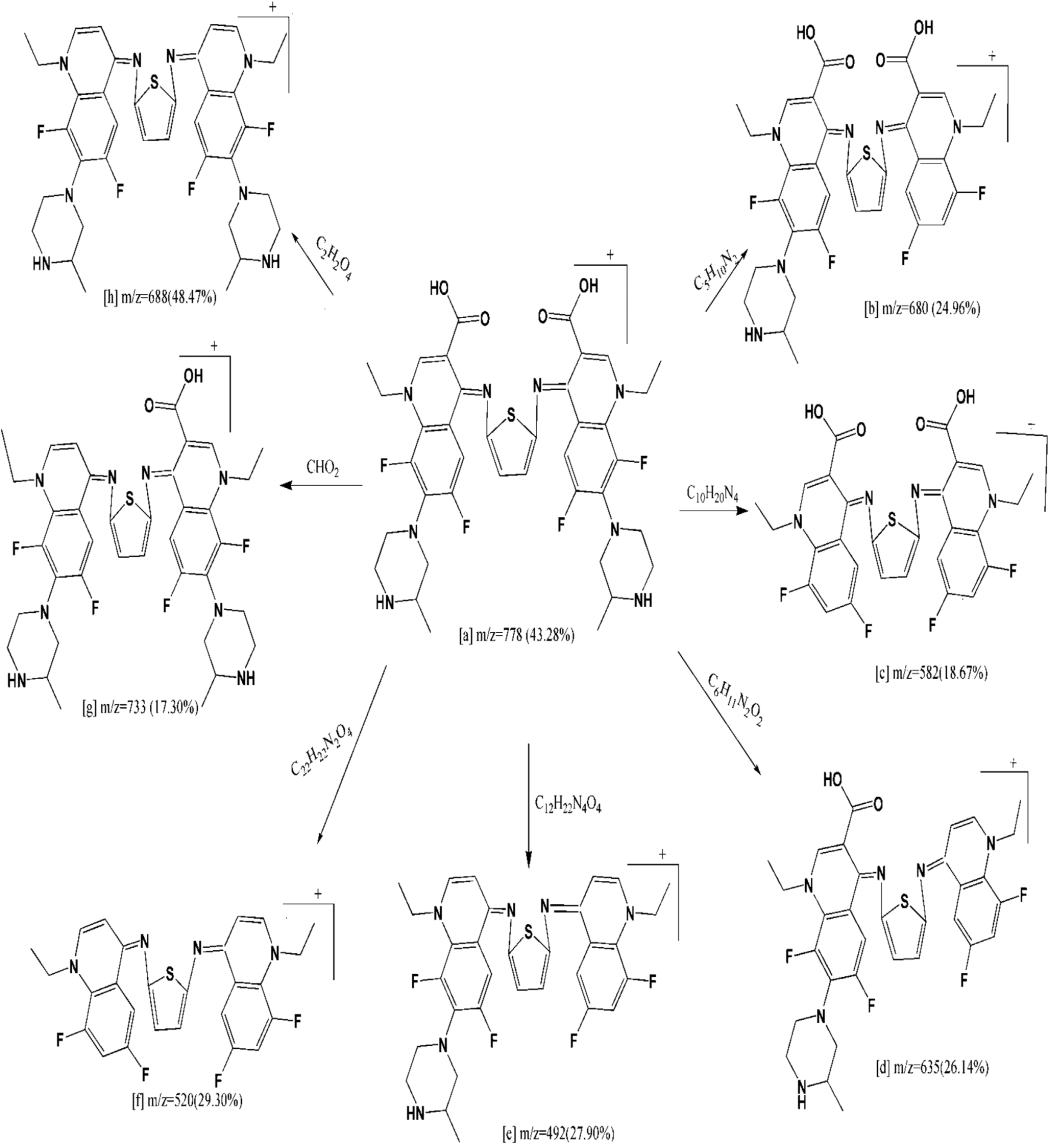
Fragmentation pattern of H_2_L.

**SCHEME 5:**
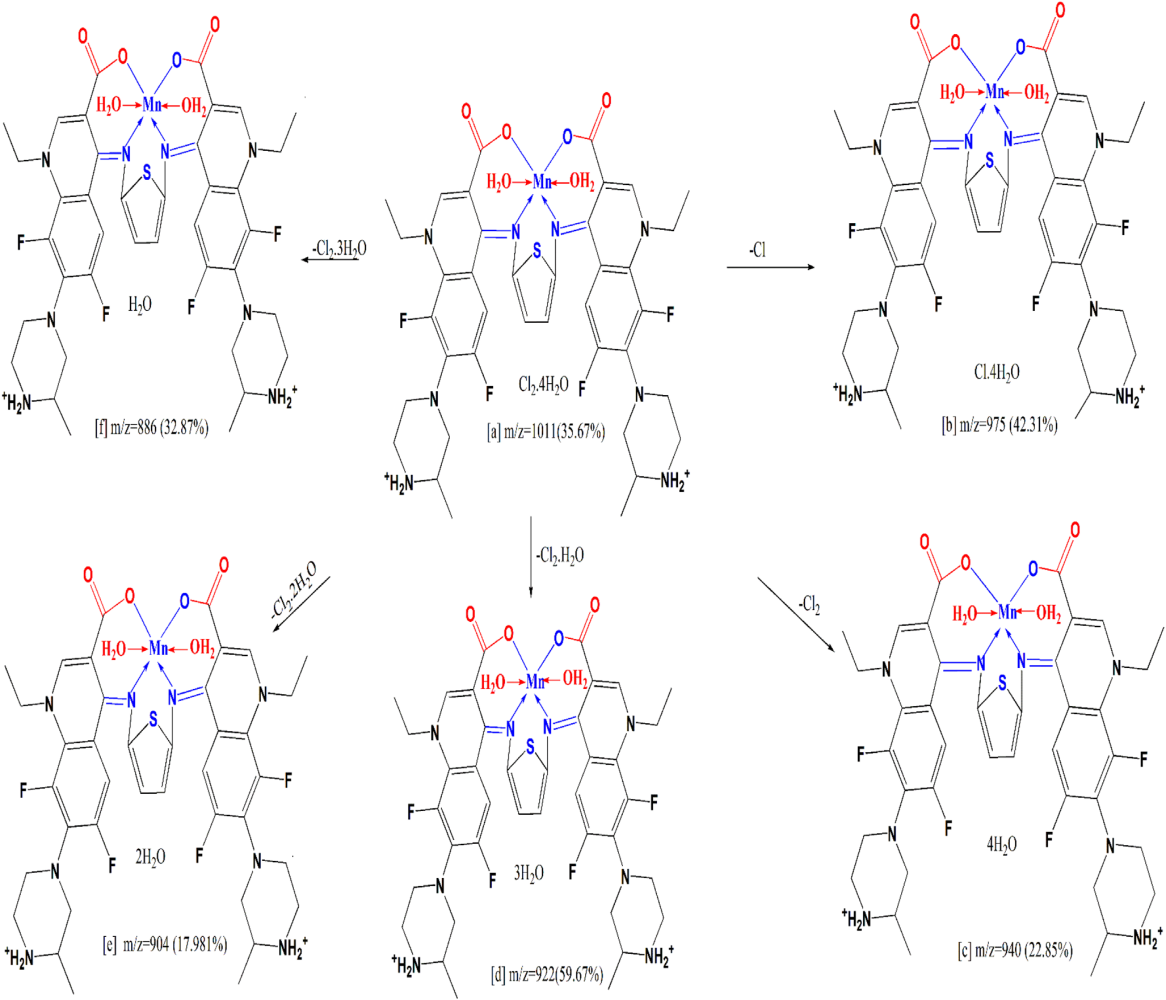
Fragmentation pattern of Complex (**2**).

Mass spectrometry proceeds by partitioning fragment ions into groups; these are subsequently multiplied by the ratio of mass to charge (*m*/*z*). The mass spectrometry of the compounds was done Figure S4 which confirmed the expected molecular formulae which will agree with the data of elemental analysis and thermogravimetric analyses. Figure S4 assigned a molecular ion peaks for H_2_L and its Complexes (**1**), (**2**) and (**3**) at *m*/*z* = 778 (43.28%), 1044 (28.12%), 1011 (35.67%), and 1033 (43.56%) respectively. The molecular ion peak [a] eliminates C_5_H_10_N_2_ to provide fragment [b] at *m*/*z* = 680 (24.96%), also it loses C_10_H_20_N_4_ to give fragment [c] at *m*/*z* = 582 (18.67%). Furthermore it loses C_6_H_11_N_2_O_2_ to give fragment [d] At *m*/*z* = 635 (26.14%), it excludes C_12_H_22_N_4_O_4_, C_22_H_22_N_2_O_4_, CHO_2_, and C_2_H_2_O_4_ resulting in fragment [e] at *m*/*z* = 492 (27.90%), [f] at *m*/*z* = 520 (29.30%), [g] at *m*/*z* = 733 (17.30%), and [h] at *m*/*z* = 688 (48.47%) (Scheme 4). A model (Scheme 5) specifies fragmentation design of Complex (**2**), the ion peak [a] at *m*/*z* = 1011 (35.67%) dissipates Cl to generate [b] at *m*/*z* = 975 (42.31%), then it removes Cl_2_ to generate [c] at *m*/*z* = 940 (22.85%). The molecular ion peak [a] dissipates Cl_2_·H_2_O for the formation of [d] at *m*/*z* = 922 (59.67%), and Cl_2_·2H_2_O produces [e] at *m*/*z* = 904 (17.98%). The molecular ion peak [a] dissipates Cl_2_·3H_2_O, yielding fragment [f] at *m*/*z* = 886 (32.87%).

### 3.6 Thermal Analysis Studies (TG and DTG)

TG and DTG investigations of the appraised H_2_L and our Complexes (1), (2), and (3) were performed (Figure S5). The TG of H_2_L progressed in three distinct phases, with a predicted elimination of mass of 96.80% (calc. 96.93%) at three maxima temperatures 225°C, 322°C, and 625°C may be accredited to the loss 2H_2_O, 14C_2_H_2_ and C_2_H_2_ + **3**C_2_N_2_ + 4HF + SO_2_ + N_2_ (Table 5). The Complex (1) shows three major degradation steps. The initial phase takes place at a maximum temperature of 60°C and leads in a weight loss of 6.55%, corresponding to the expulsion of four molecules of uncoordinated water [63, 64, 65]. The second phase in deterioration proceeds at *T*_max_ 296°C with weight loss of 32.26% (calc. 32.37), corresponding to loss of 13C_2_H_2_. The third stage of breakdown occurs at two maxima of 595°C and 703°C, resulting in a weight diminished by 44.89% (calc. 44.27), which corresponds to C_2_H_2_ + 2NH_3_ + 4HF + 3HCl + 0.5H_2_O + 2NO + **C_2_** N_2_ + SO_2_ + **N_2_**. The actual weight loss from these three procedures is 83.70%, which is similar to the calculated find of 83.53% for a final product containing 0.5Cr_2_O_3_ and 8C. TG Complexes (**2**) and (**3**) decompose in three phases and exhibit essentially comparable thermal behavior: The first one displays the disintegration of lattice water molecules at *T*_max_ 114°C and 106°C [65]. The second decomposition step at *T*_max_ 314°C and 306°C displayed an elimination of 16C_2_H_2_ 40.94% (calc. 41.11%) and 14C_2_H_2_ 35.00% (calc. 35.21%), respectively. The third step requires breakdown at 598°C and 573°C maxima revealed to loss of 2C_2_H_2_ + 4HF + 2HCl + 0.5H_2_ + 2NO_2_ + SO_2_ + C_2_N_2_ + 2N_2_ 46.56% (calc. 46.34%) and 4C_2_H_2_ + 2NH_3_ + 4HF + 2HCl + 0.5H_2_O + 2NO_2_ + SO_2_ + C_2_N_2_ + 2N_2_ 50.80% (calc. 50.38%), respectively, leaving Mn and Co as a residues (Table 5). The stability of the complexes is different which depending on the strength of the bonds between donor atom of the ligand with metal ions and strength of the inter molecular hydrogen bond which give the different stability of one complex to the other. Complex **2** is more stable than other.

**TABLE 5:** The maximum temperature *T*_max_ (°C) and weight loss values of the decomposition stages for H_2_L and its metal complexes.

Compounds	Decomposition	*T*_max_ (°C)	Loss weight (%)		Lost species
			Calc.	Found	
H_2_L	First step	225	4.63	4.55	2H_2_O
	Second step	322	46.79	46.56	14C_2_H_2_
	Third step	625	45.50	45.69	C_2_H_2_ + 3C_2_N_2_ + 4HF + SO_2_ + N_2_
	Total loss		96.92	96.80	
	Residue		3.08	3.20	2C
(**1**)	First step	60	6.89	6.55	4H_2_O
	Second step	296	32.37	32.26	13C_2_H_2_
	Third step	595, 703	44.27	44.89	C_2_H_2_ + 2NH_3_ + 4HF + 3HCl + 0.5H_2_O + 2NO + C_2_N_2_ + SO_2_ + N_2_
	Total loss		83.53	83.70	
	Residue		16.47	16.30	0.5Cr_2_O_3_ + 8C
(**2**)	First step	114	7.12	7.05	4H_2_O
	Second step	314	41.11	40.94	16C_2_H_2_
	Third step	598	46.34	46.56	2C_2_H_2_ + 4HF + 2HCl + 0.5H_2_ + 2NO_2_ + SO_2_ + C_2_N_2_ + 2N_2_
	Total loss		94.57	94.55	
	Residue		5.43	5.45	Mn
(**3**)	First step	106	8.71	8.65	5H_2_O
	Second step	306	35.21	35.00	14C_2_H_2_
	Third step	573	50.38	50.80	4C_2_H_2_ + 2NH_3_ + 4HF + 2HCl + 0.5H_2_O + 2NO_2_ + SO_2_ + C_2_N_2_ + 2N_2_
	Total loss		94.30	94.45	
	Residue		5.70	5.55	Co

### 3.7 Calculation of Thermodynamic Parameters

Coats–Redfern and Horowitz–Metzger [66, 67] were employed to investigate the kinetic thermodynamic attributes associated with thermal breakdown for our compounds, including activation energy (*E*_a_), enthalpy (Δ*H*), entropy (Δ*S*), and Gibbs free energy (ΔG*) using equations (5‐8) (Figure S6).

(5) $$\ln\;X=\ln\left[\frac{-\ln\left(1-\alpha \right)}{T^{2}}\right]=\frac{-E*}{RT}+\ln\left[\frac{AR}{\varphi E*}\right]\;\mathrm{for}\;n=1$$

(6) $$\ln\;X=\ln\left[\frac{-\ln{\left(1-\alpha \right)}^{1nn}}{T^{2}\left(1nn\right)}\right]=\frac{-E*}{RT}+\ln\left[\frac{AR}{\varphi E*}\right]\;\mathrm{for}\;n\neq 1$$

(7) $$\ln[-\ln(1-\alpha )]=\frac{E_{a}\theta }{RT_{s}^{2}}\;\;\;\;\;\;\;\;\;\mathrm{for}\;n=1$$

(8) $$\ln\left[\frac{\left\{1-{\left(1-\alpha \right)}^{1-n}\right\}}{\left(1-n\right)}\right]=\ln(\frac{A}{\Phi }\frac{RT_{s}^{2}}{E})-\frac{E_{a}}{RT_{s}}+\frac{E_{a}\theta }{RT_{s}^{2}}\;\;\mathrm{for}\;n\neq 1\;\;\;\;\;\;\;$$

For H_2_L and its metal complexes, the *E*_a_ of decomposition ranged from 12.89 to 90.06 kJ mol^−1^ in Coats–Redfern model and 15.10–57.45 kJ mol^−1^ in Horowitz–Metzger model (Table 6 and Figure S6). The high (*E*_a_) values illustrate the complexes' thermal stability [68]. An increasing ΔG* value signifies that the subsequent ligand will be removed at more gradually than the subsequent ligand, resulting in an increase in *T*ΔS* from one stage to the next. This may be ascribed to the structural rigidity of the residual complex after the expulsion of one or more ligands, as opposed to the previous complex, which requires more energy, *T*ΔS*, for its rearrangement before experiencing any change. The negative values for ΔS* in all complexes indicated their stability [66], also positive values for ΔH* indicate endothermic breakdown processes [31, 32, 68].

**TABLE 6:** Thermal behavior and kinetic parameters of H_2_L and its metal complexes determined using the Coats–Redfern (CR) and Horowitz–Metzger (HM) methods.

Compounds	Decomposition range (K)	*T*_s_ (K)	Method	Parameters					*r*	SD
				E* (kJ/mol)	*A* (s^−1^)	ΔS* (kJ/mol K)	ΔH* (kJ/mol)	ΔG* (kJ/mol)		
H2L	540–752	595	CR	12.89	0.1555	−0.2661	7.95	166.29	0.990	0.118
			HM	57.45	3.85 × 10^2^	−0.2011	52.50	172.18	0.972	0.205
(**1**)	311–339	326	CR	25.95	2.5667	−0.2378	23.24	100.76	0.984	0.130
			HM	23.66	27.506	−0.2180	20.95	92.04	0.974	0.168
	486–671	569	CR	41.16	3.8223	−0.2391	36.43	172.50	0.982	0.152
			HM	44.60	34.205	−0.2209	39.87	165.56	0.968	0.200
(**2**)	300–360	323	CR	19.28	0.1908	−0.2593	16.59	36.100	0.987	0.117
			HM	17.42	2.1940	−0.239	14.74	91.95	0.974	0.168
	448–733	587	CR	45.36	12.858	−0.2293	40.48	175.08	0.984	0.142
			HM	53.88	1.14 × 10^5^	−0.1539	49.01	139.35	0.973	0.184
(**3**)	301–353	327	CR	30.77	2.678	−0.2374	28.05	105.71	0.985	0.141
			HM	15.10	0.7296	−0.2482	12.38	93.57	0.970	0.198
	407–688	579	CR	90.06	4.71 × 10^3^	−0.1801	85.25	189.53	0.988	0.142
			HM	53.73	2.25 × 10^2^	−0.2053	48.92	167.84	0.980	0.181

*Note*: Correlation coefficients of the Arrhenius plots (*r*) and standard deviation (SD).

### 3.8 Antibacterial and Antifungal Activities

The antimicrobial potential of the synthesized H_2_L and its metal complexes was assessed against four pathogenic bacterial strains—*B. cereus* and *S. aureus* (Gram‐positive), and *E. coli* and *P. aeruginosa* (Gram‐negative)—as well as two fungal species, *C. albicans* and *P. vulpinum* (Figure S7). The antimicrobial efficacy was benchmarked against standard reference antibiotics: ciprofloxacin and amikacin for bacteria, and clotrimazole for fungi. Ethylenediamine and DMF were used as negative controls to confirm the specificity of antimicrobial action. The antimicrobial activity was quantified by calculating the activity index (%) relative to the inhibition zones produced by the standard drugs, as summarized in Table 7 and illustrated in Figure S8 and S9. Among the tested compounds, Complex (**1**) demonstrated the highest bactericidal efficiency, showing very strong activity against *E. coli* (73.07%) and *S. aureus* (70.83%), and moderate activity against *P. aeruginosa* (43.47%), *C. albicans* (48.14%), and *P. vulpinum* (33.33%). Complex (**2**) exhibited notable inhibitory effects against *E. coli* (68.75%), *S. aureus* (58.33%), and *B. cereus* (65.21%). Meanwhile, Complex (**3**) displayed strong antifungal activity against *P. aeruginosa* (73.91%) and *C. albicans* (66.66%), along with moderate activity against *E. coli* (42.30%), *B. cereus* (60.86%), and *P. vulpinum* (40.74%). These findings suggest that chelation significantly enhances the antimicrobial properties of H_2_L. The observed effects are likely attributed to the chelation‐mediated reduction in the metal ion's polarity through electron delocalization over the aromatic system, increasing lipophilicity. This property facilitates penetration of the complexes through microbial cell membranes, in line with the overtone concept and Tweedy's chelation theory [69, 70, 71, 72]. Based on the activity indices, Complex (**1**) exhibited the highest overall antimicrobial efficacy among the synthesized compounds.

**TABLE 7:** Antimicrobial activity of H_2_L and its metal complexes.

Compound	E. coli		P. aeruginosa		S. aureus		B. cereus		C. albicans		P. vulpinum	
	Diameter of inhibition zone (mm)	% Activity index	Diameter of inhibition zone (mm)	% Activity index	Diameter of inhibition zone (mm)	% Activity index	Diameter of inhibition zone (mm)	% Activity index	Diameter of inhibition zone (mm)	% Activity index	Diameter of inhibition zone (mm)	% Activity index
**H_2_L**	9 ± 0.3	34.61	10 ± 0.15	65.21	11 ± 0.07	45.83	12 ± 0.26	52.17	11 ± 0.21	40.74	8 ± 0.05	48.14
**(1)**	19^+2^ ± 0.2	73.07	14^+1^ ± 0.3	58.33	17^+2^ ± 0.17	70.83	10 ± 0.29	43.47	13^+1^ ± 0.11	48.14	10^+1^ ± 0.16	33.33
**(2)**	16^+2^ ± 0.4	68.75	8 ± 0.25	34.75	14^+1^ ± 0.38	58.33	15^+1^ ± 0.17	65.21	8 ± 0.19	59.25	6 ± 0.12	22.22
**(3)**	11^+1^ ± 0.1	42.30	17^+2^ ± 0.66	73.91	9 ± 0.11	37.50	14^+1^ ± 0.12	60.86	18^+2^ ± 0.46	66.66	11^+1^ ± 0.23	40.74
**Ciprofloxacin**	26 ± 0.1	100	23 ± 0.29	100	24 ± 0.27	100	23 ± 0.33	100	NA	—	NA	—
**Clotrimazole**	NA	—	NA	—	NA	—	NA	—	27 ± 0.52	100	27 ± 0.45	100
**Amikacin**	26 ± 0.1	100	23 ± 0.3	100	24 ± 0.27	100	23 ± 0.33	100	NA		NA	
**Ethylene diamine**	NA	—	NA	—	NA	—	NA	—	NA	—	NA	—
**DMF**	NA	—	NA	—	NA	—	NA	—	NA	—	NA	—

*Note*: The activity was evaluated by measuring the diameter of the inhibition zones (in millimeters, mm) and the associated activity index was also calculated. Statistical significance was assessed using the paired Student's *t*‐test. Results were interpreted as follows: *p* ^ns^—not significant (*p* > 0.05); *p* ^+1^—significant (*p* < 0.05); *p* ^+2^—highly significant (*p* < 0.01); *p* ^+3^—very highly significant (*p* < 0.001).

Abbreviation: NA, no activity.

### 3.9 Cytotoxic Evaluation

To investigate the potential anticancer properties of the synthesized Schiff base ligand (H_2_L) and its metal complexes, their cytotoxic effects were evaluated in vitro using a normal human prostate epithelial cell line (PrEC). Cytotoxicity was assessed via the MTT assay, which measures mitochondrial dehydrogenase activity as an indicator of cell viability. PrEC cells were treated with increasing concentrations (12.5, 25, 50, 100, and 200 µM) of each compound for 48 h. Following treatment, the formation of purple formazan crystals from MTT reduction by viable cells was quantified spectrophotometrically at 570 nm. The resulting cell viability data were used to determine IC_50_ values, as shown in Table 8. The results demonstrated that all tested compounds, including the free ligand (H_2_L) and its metal complexes, exhibited significant cytotoxicity against normal PrEC cells. Notably, even the lowest concentration tested (12.5 µM) induced marked reductions in cell viability. Among the compounds, Complex **3** displayed the greatest cytotoxic potential, with the lowest IC_50_ value of 6.30 µM, followed by Complex (**1**) (7.32 µM), Complex (**2**) (8.30 µM), and H_2_L (10.43 µM). These findings indicate that the cytotoxic effects are enhanced upon metal coordination, likely due to increased cellular uptake and reactivity. However, the high cytotoxicity observed in non‐malignant cells presents a significant limitation for the development of these compounds as anticancer agents. The lack of selectivity toward cancerous versus normal cells suggests a narrow therapeutic window. Therefore, despite their promising antimicrobial efficacy, the current forms of H_2_L and its metal complexes are not suitable for oncology applications without further structural modifications aimed at enhancing tumor‐specific targeting and reducing off‐target cytotoxicity.

**TABLE 8:** Cytotoxic Effects of H_2_L Schiff base and its metal complexes on normal human prostate epithelial cells (PrEC): IC_50_values.

Compounds	IC_50_ (µM) ± SE
H_2_L Schiff base	10.43 ± 0.014
Complex **1**	7.32 ± 0.12
Complex **2**	8.3 ± 0.016
Complex **3**	6.3 ± 0.17

The half‐maximal inhibitory concentration (IC_50_) values, along with their respective standard errors (SE), were calculated using nonlinear regression analysis of dose–response curves generated from the MTT assay. Normal prostate epithelial cells (PrEC) were treated with increasing concentrations (12.5, 25, 50, 100, and 200 µM) of the test compounds for 48 h. Cell viability was quantified based on the metabolic reduction of MTT to formazan, and IC_50_ values were derived at a 95% confidence interval.

## 4 Conclusion

In this study, a novel tetradentate Schiff base ligand (H_2_L) was synthesized by condensing thiophene‐2,5‐diamine with lomefloxacin and then complexed with chromium(III), manganese(II), and cobalt(II) ions. Spectroscopic analyses (UV–Vis, magnetic susceptibility) confirmed octahedral coordination geometry, and FTIR data indicated coordination via azomethine nitrogen and carboxylate oxygen atoms. Thermal analyses (TG/DTG) and kinetic modelling (Coats–Redfern, Horowitz–Metzger) showed high thermal stability and endothermic decomposition. Antimicrobial assays revealed notable activity for all compounds, with the chromium(III) complex being most effective, followed by cobalt(II) and manganese(II), indicating metal coordination enhances bioactivity. However, MTT cytotoxicity assays on normal human prostate epithelial cells (PrEC) showed significant toxicity at low concentrations (12.5 µM). Complex **3** had the highest cytotoxicity (IC_50_ = 6.30 µM), followed by Complex **1**, Complex **2**, and H_2_L. These results indicate poor selectivity between normal and malignant cells, limiting therapeutic potential. Although the Schiff base and its metal complexes demonstrate strong antimicrobial activity and thermal stability, their high cytotoxicity toward normal cells is a major drawback. Future efforts should focus on modifying the ligand structure or employing targeted delivery systems to enhance selectivity and reduce off‐target toxicity, aiming for safer and more effective therapeutic agents.

## Author Contributions

**Amira A. Mohamed**: conceptualization, formal analysis, biological investigations, validation, writing – original draft preparation. **Mohammed S. El‐Gedamy**: formal analysis, biological investigations, writing – review and editing. **Sadeek A. Sadeek**: conceptualization, biological investigations, supervision, validation, writing – original draft preparation, writing – review and editing. **Hazem S. Elshafie**: formal analysis, supervision, validation, writing – original draft preparation.

## Ethics Statement

The authors have nothing to report.

## Consent

The authors have nothing to report.

## Conflicts of Interest

The authors declare no conflicts of interest.

## Supporting information




**Supporting File 1**: cbdv70504‐sup‐0001‐SuppMat.pdf. **Figure S1**: Infrared spectra for **H_2_L** and its metal complexes; **Figure S2**: Electronic absorption spectra for **H_2_L** and its metal complexes.; **Figure S3** ^1^H NMR spectra for **H_2_L** and its metal complexes., **Figure S4** Mass spectra diagrams for **H_2_L** and its metal complexes, **Figure S5** TG and DTG diagrams for **H_2_L** and its metal complexes, **Figure S6** The diagrams of kinetic parameters of **H_2_L** and its metal complexes, **Figure S7** Illustrates the antimicrobial efficacy of **H_2_L** and its metal complexes, presented as mean inhibition zones (mm). Error bars represent the standard error (SE), indicating data variability and reproducibility, **Figure S8** Activity index for bacteria strains of **H_2_L** and its metal complexes, **Figure S9** Activity index for fungi strains of **H_2_L** and its metal complexes.

## Data Availability

The authors have nothing to report.
