# Supplementary material for: First Report on some N2O2‐Donor Sets Tetradentate Schiff Base and Its Metal Complexes: Characterization and Antimicrobial Investigation
Source: Chem Biodivers. 2025 Sep 22;22(12):e01117. doi: 10.1002/cbdv.202501117 (PMC12716001; doi:10.1002/cbdv.202501117)
Supplement: Supplementary file 1 — Supporting File 1: cbdv70504‐sup‐0001‐SuppMat.pdf. Figure S1: Infrared spectra for H2L and its metal complexes; Figure S2: Electronic absorption spectra for H2L and its metal complexes.; Figure S3 1H NMR spectra for H2L and its metal complexes., Figure S4 Mass spectra diagrams for H2L and its metal complexes, Figure S5 TG and DTG diagrams for H2L and its metal complexes, Figure S6 The diagrams of kinetic parameters of H2L and its metal complexes, Figure S7 Illustrates the antimicrobial efficacy of H2L and its metal complexes, presented as mean inhibition zones (mm). Error bars represent the standard error (SE), indicating data variability and reproducibility, Figure S8 Activity index for bacteria strains of H2L and its metal complexes, Figure S9 Activity index for fungi strains of H2L and its metal complexes. [file CBDV-22-e01117-s001.pdf]

Supporting Information

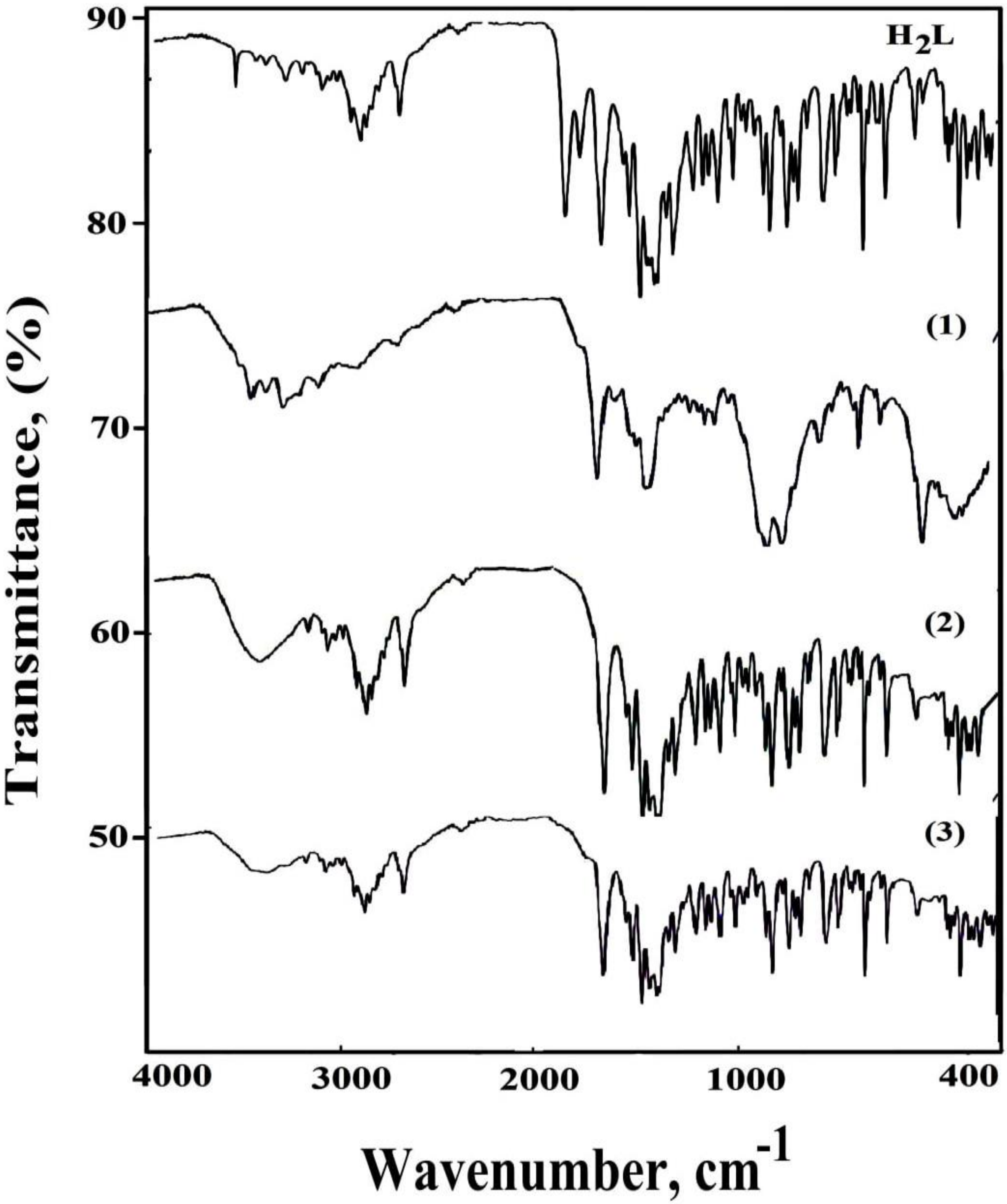

Figure S1 Infrared spectra for **H_2L** and its metal complexes.

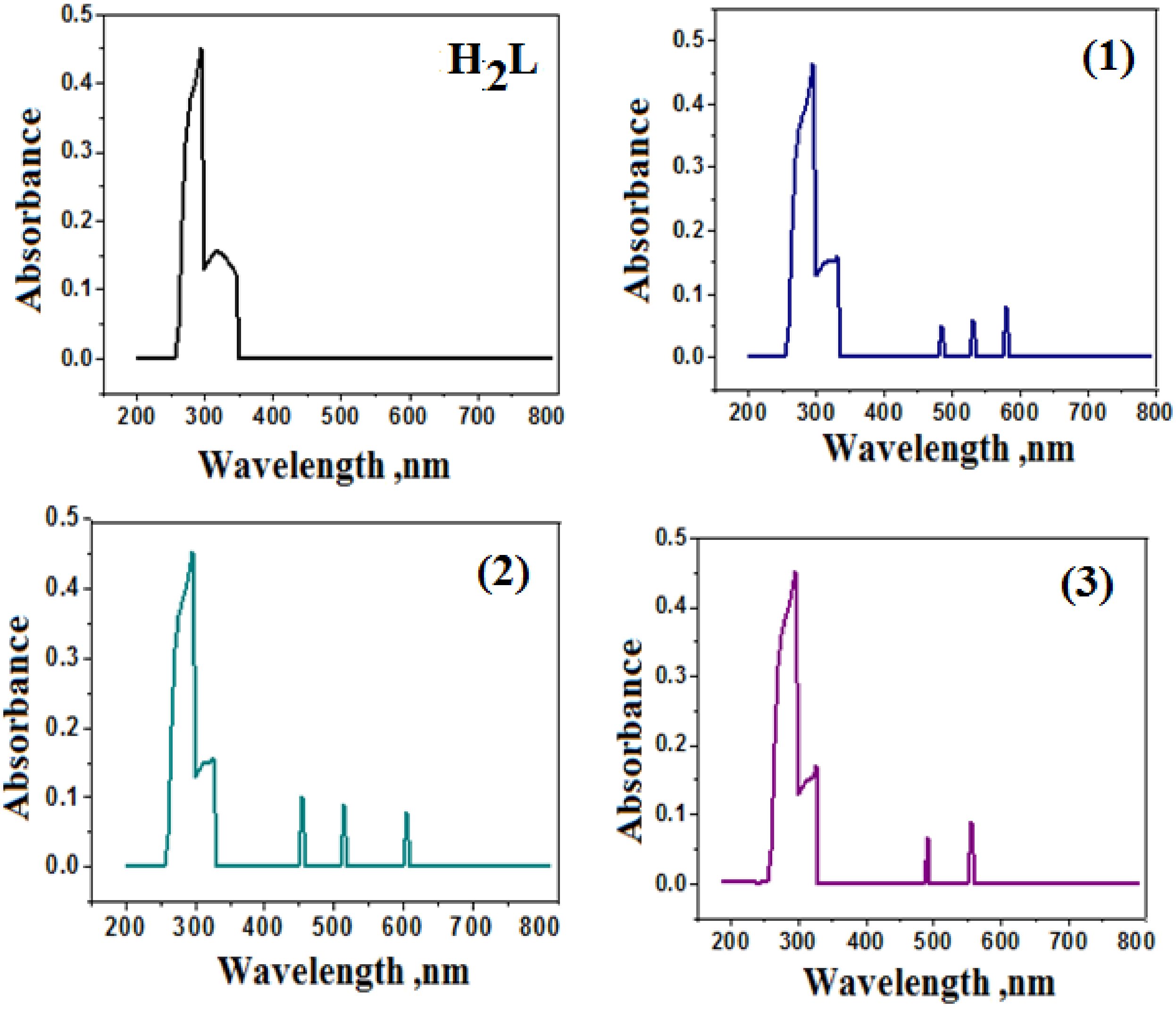

Figure S2 Electronic absorption spectra for **H_2L** and its metal complexes.

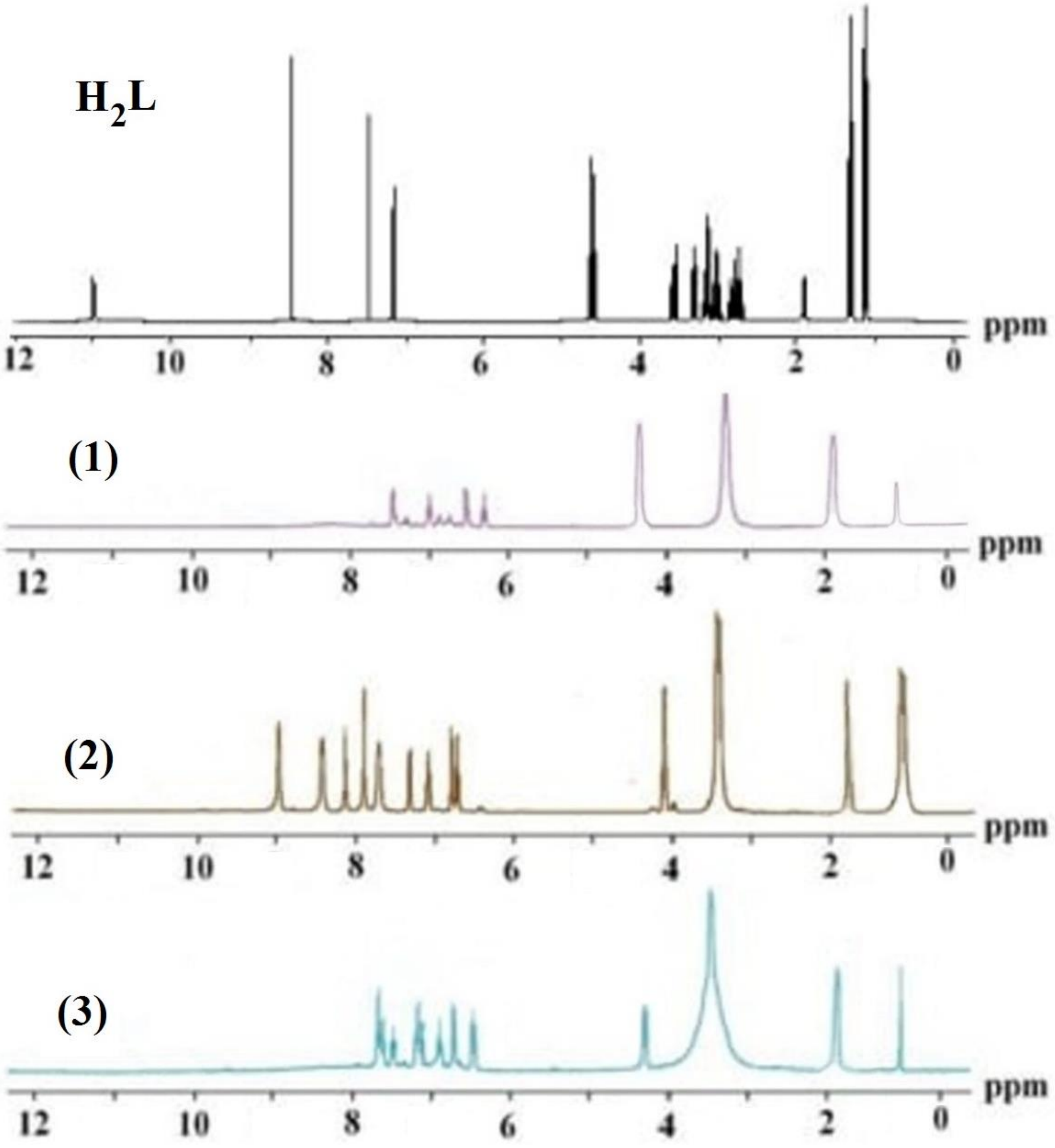

Figure S3 ^{1}H NMR spectra for **H_2L** and its metal complexes.

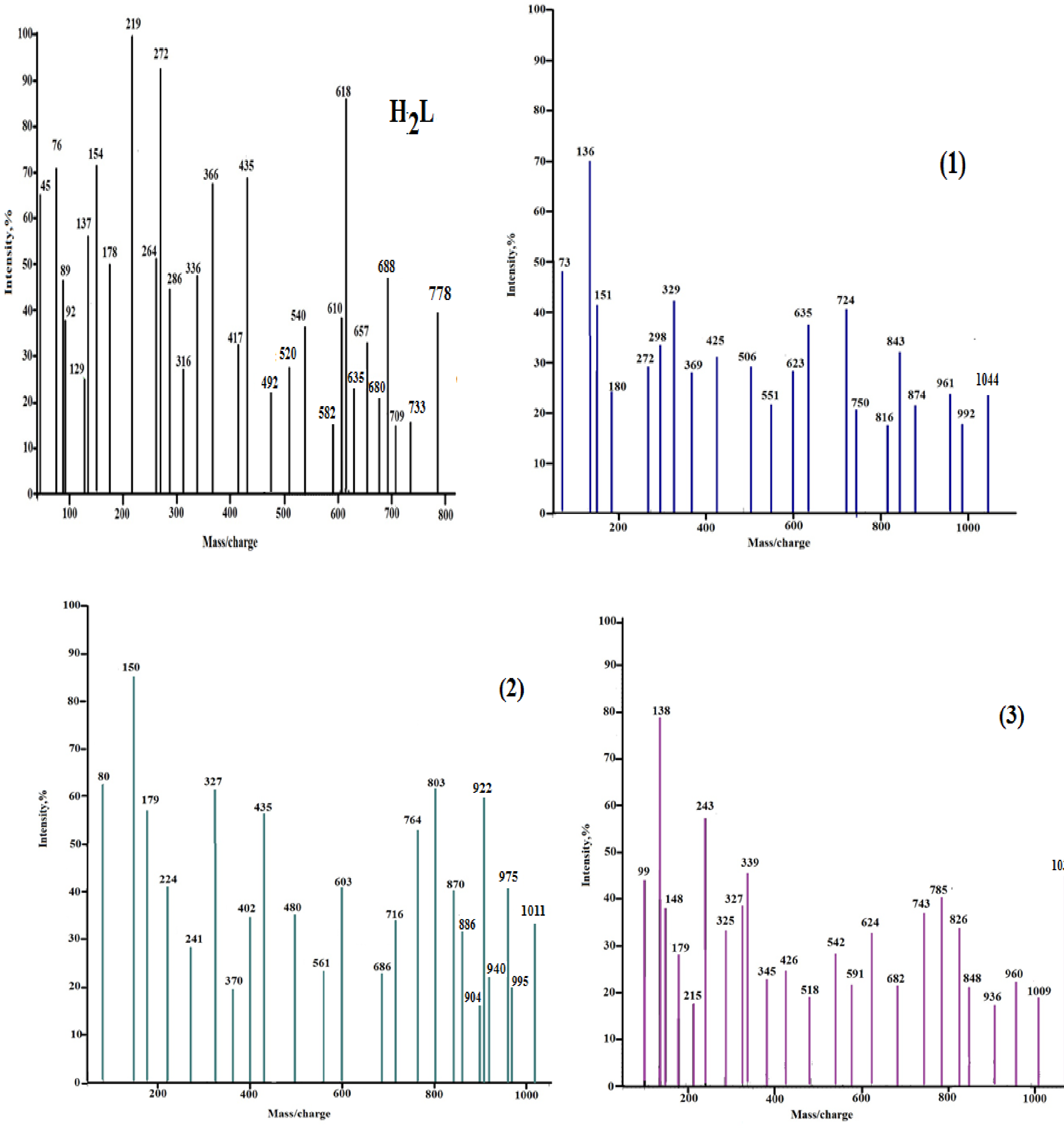

Figure S4 Mass spectra diagrams for $\mathbf{H_2L}$ and its metal complexes.

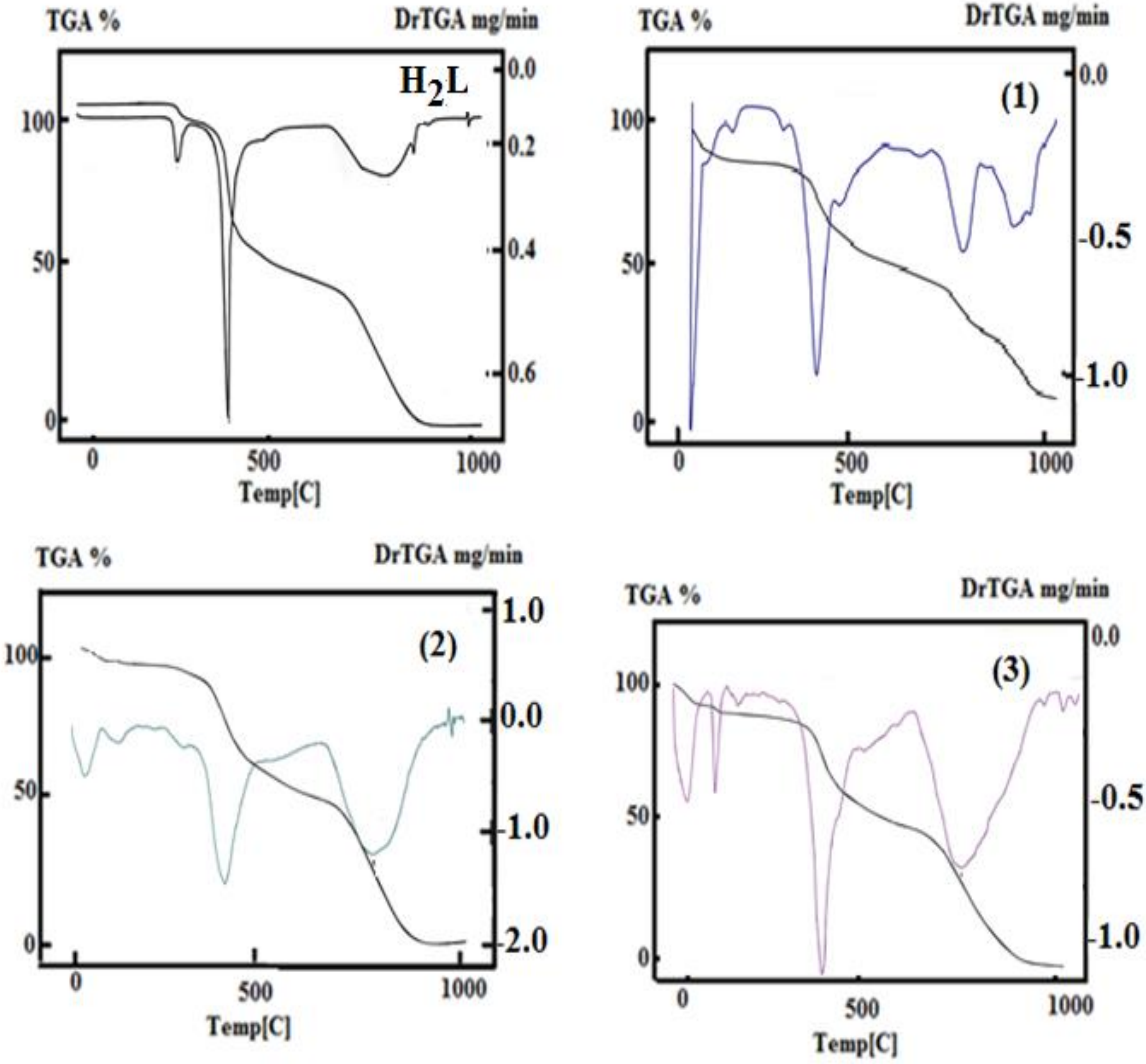

Figure S5 TG and DTG diagrams for **H_2L** and its metal complexes**.**

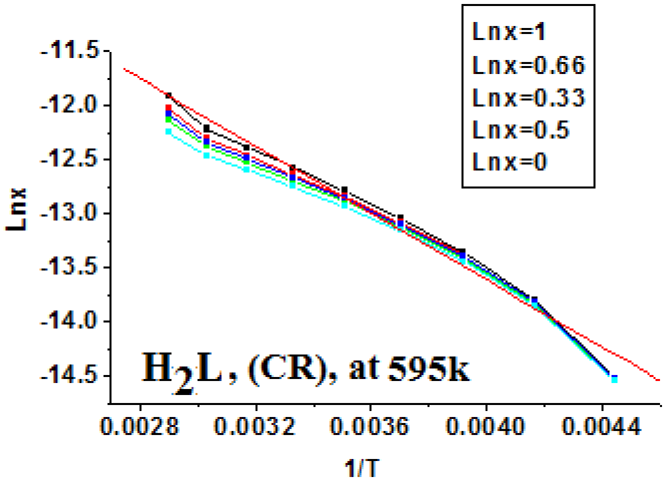
Lnx=1
Lnx=0.66
Lnx=0.33
Lnx=0.5
Lnx=0
H2L, (CR), at 595k
Lnx
1/T

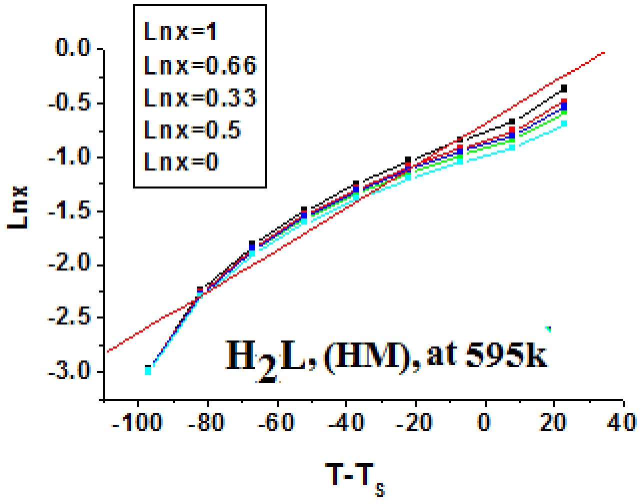
Lnx=1
Lnx=0.66
Lnx=0.33
Lnx=0.5
Lnx=0
H2L, (HM), at 595k
Lnx
T-Ts

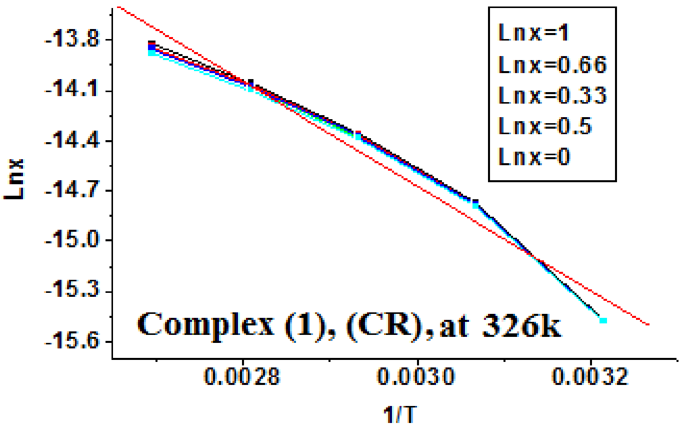
Lnx=1
Lnx=0.66
Lnx=0.33
Lnx=0.5
Lnx=0
Complex (1), (CR), at 326k
Lnx
1/T

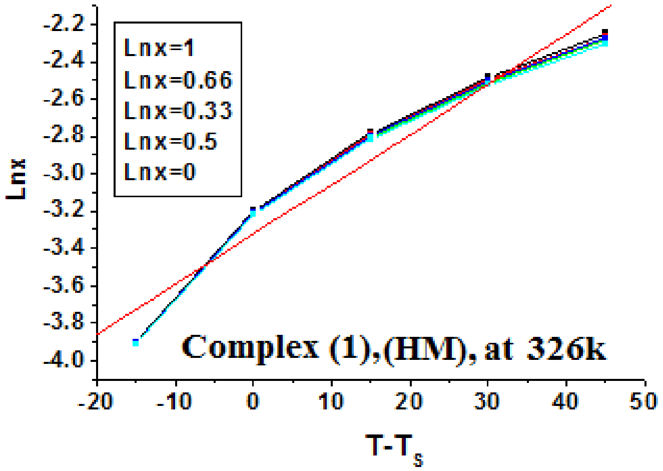
Lnx=1
Lnx=0.66
Lnx=0.33
Lnx=0.5
Lnx=0
Complex (1),(HM), at 326k
Lnx
T-Ts

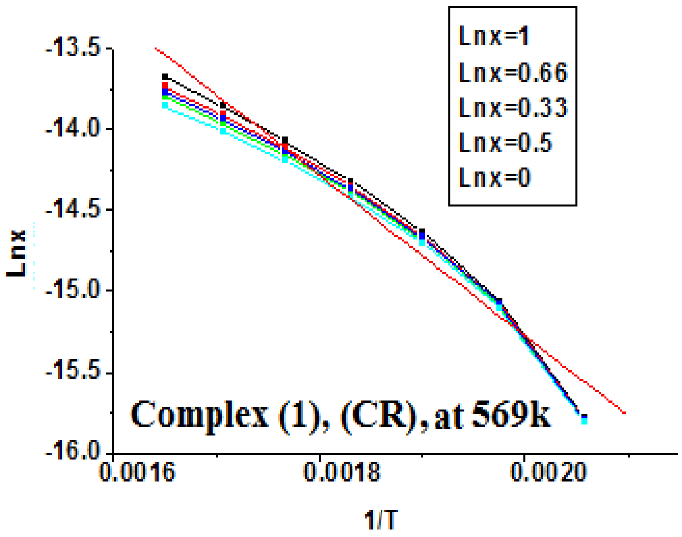
Lnx=1
Lnx=0.66
Lnx=0.33
Lnx=0.5
Lnx=0
Complex (1), (CR), at 569k
Lnx
1/T

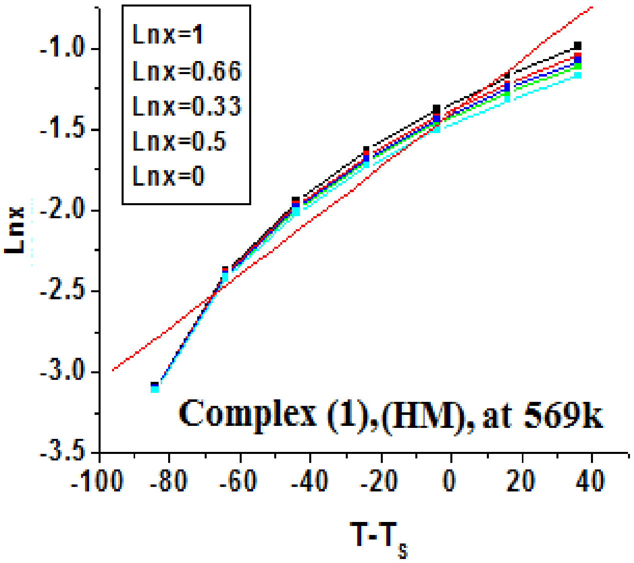
Lnx=1
Lnx=0.66
Lnx=0.33
Lnx=0.5
Lnx=0
Complex (1),(HM), at 569k
Lnx
T-Ts

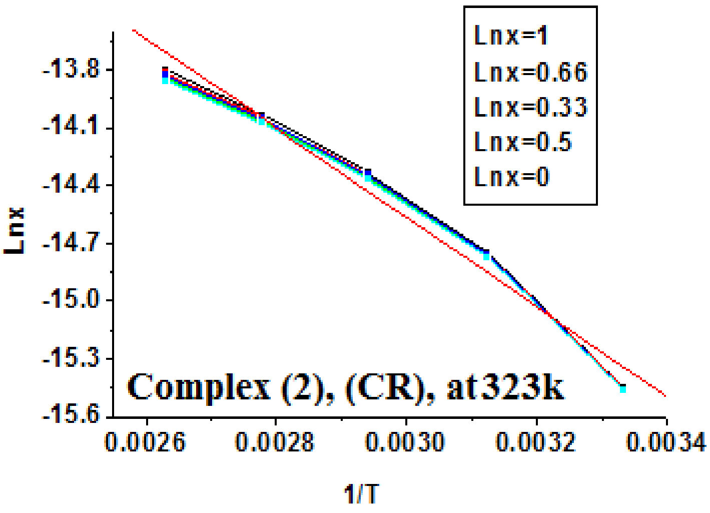
Lnx=1
Lnx=0.66
Lnx=0.33
Lnx=0.5
Lnx=0
Complex (2), (CR), at323k
Lnx
1/T
-13.8
-14.1
-14.4
-14.7
-15.0
-15.3
-15.6
0.0026
0.0028
0.0030
0.0032
0.0034

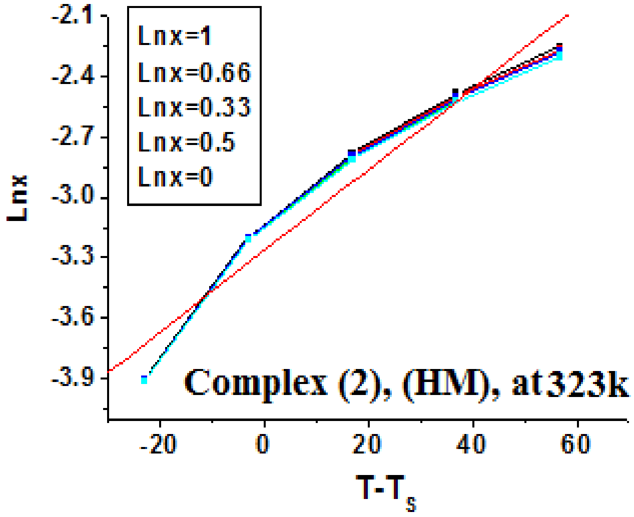
Lnx=1
Lnx=0.66
Lnx=0.33
Lnx=0.5
Lnx=0
Complex (2), (HM), at323k
Lnx
T-T$_s$
-2.1
-2.4
-2.7
-3.0
-3.3
-3.6
-3.9
-20
0
20
40
60

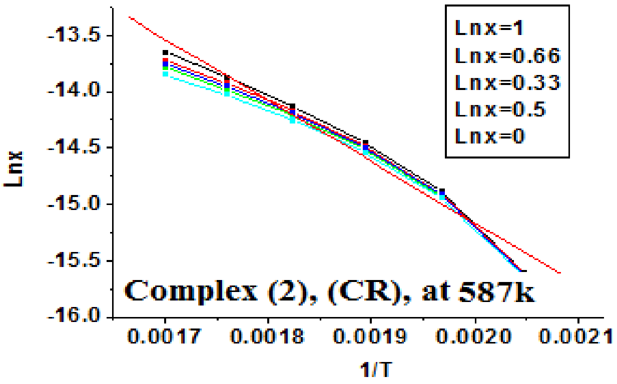
Lnx=1
Lnx=0.66
Lnx=0.33
Lnx=0.5
Lnx=0
Complex (2), (CR), at 587k
Lnx
1/T
-13.5
-14.0
-14.5
-15.0
-15.5
-16.0
0.0017
0.0018
0.0019
0.0020
0.0021

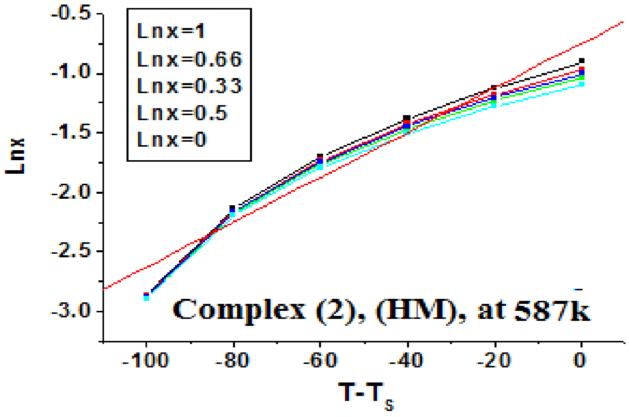
Lnx=1
Lnx=0.66
Lnx=0.33
Lnx=0.5
Lnx=0
Complex (2), (HM), at 587k
Lnx
T-T$_s$
-0.5
-1.0
-1.5
-2.0
-2.5
-3.0
-100
-80
-60
-40
-20
0

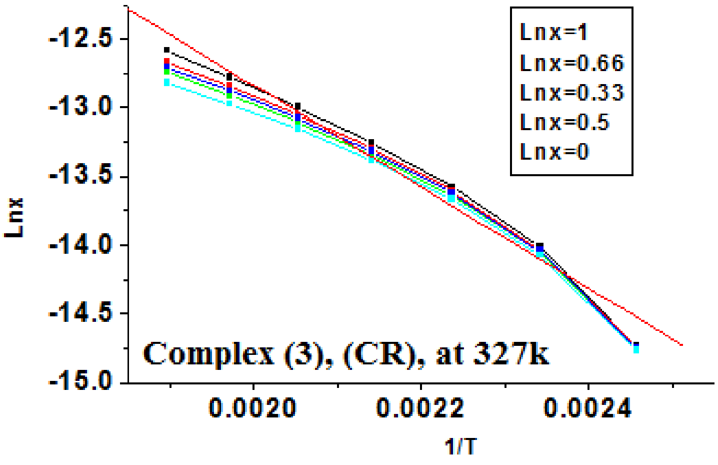
Lnx=1
Lnx=0.66
Lnx=0.33
Lnx=0.5
Lnx=0
Complex (3), (CR), at 327k
Lnx
1/T
-12.5
-13.0
-13.5
-14.0
-14.5
-15.0
0.0020
0.0022
0.0024

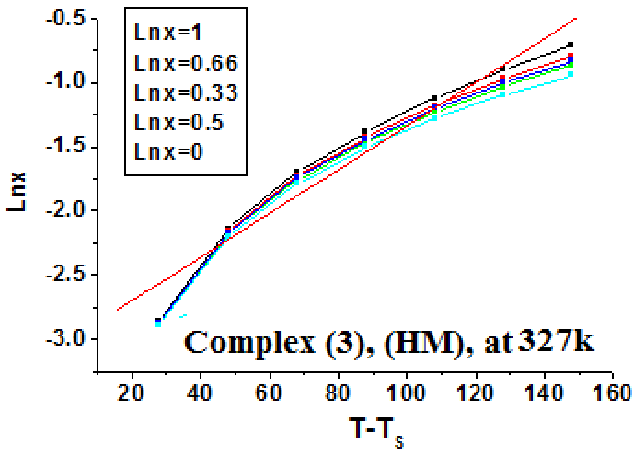
Lnx=1
Lnx=0.66
Lnx=0.33
Lnx=0.5
Lnx=0
Complex (3), (HM), at327k
Lnx
T-T$_s$
-0.5
-1.0
-1.5
-2.0
-2.5
-3.0
20
40
60
80
100
120
140
160

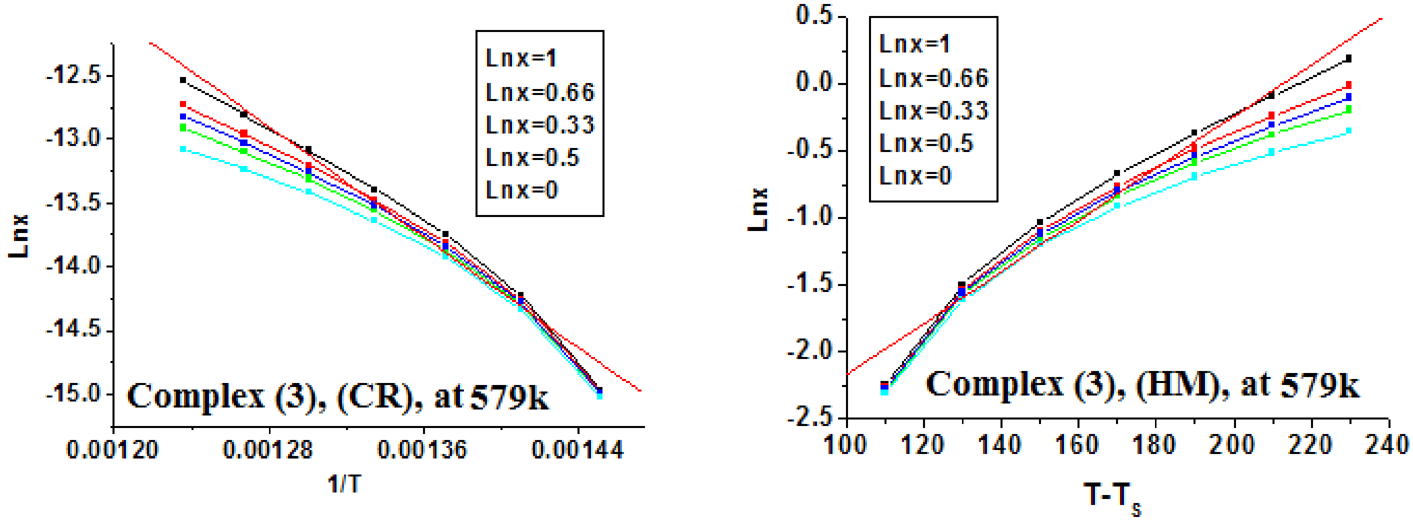

Figure S6 The diagrams of kinetic parameters of $\mathbf{H_2L}$ and its metal complexes.

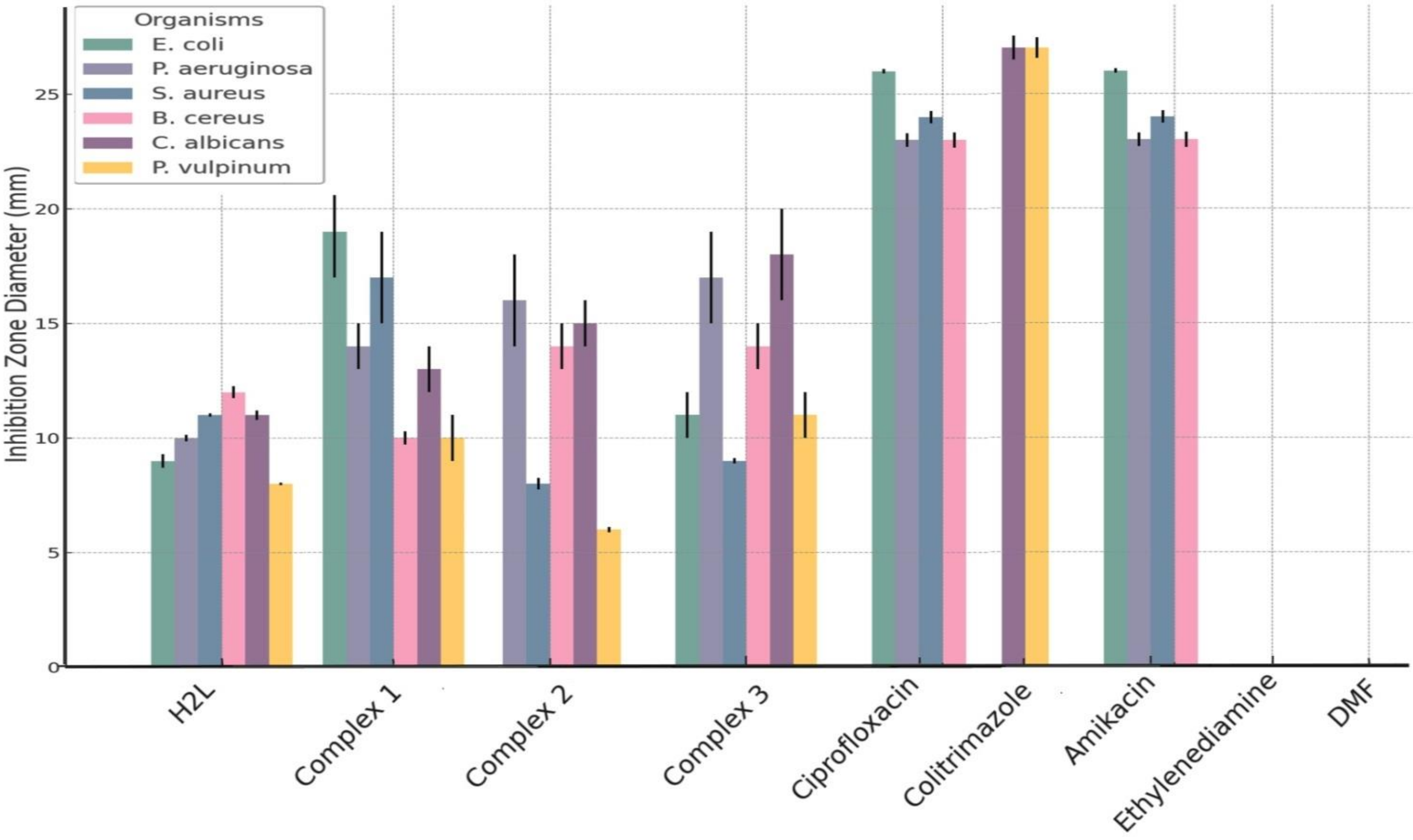

Figure S7 Illustrates the antimicrobial efficacy of $\mathbf{H_2L}$ and its metal complexes, presented as mean inhibition zones (mm). Error bars represent the standard error (SE), indicating data variability and reproducibility

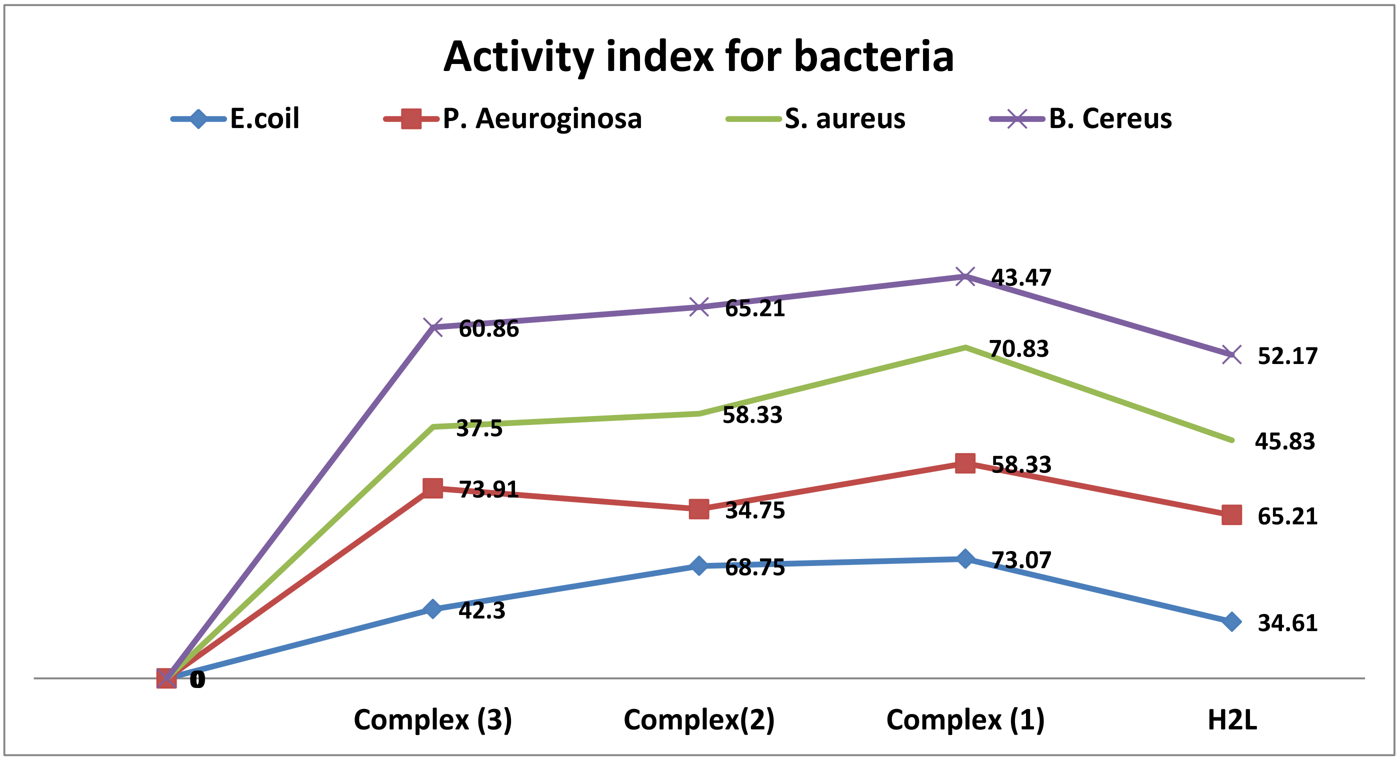

Figure S8 Activity index for bacteria strains of **H_2L** and its metal complexes.

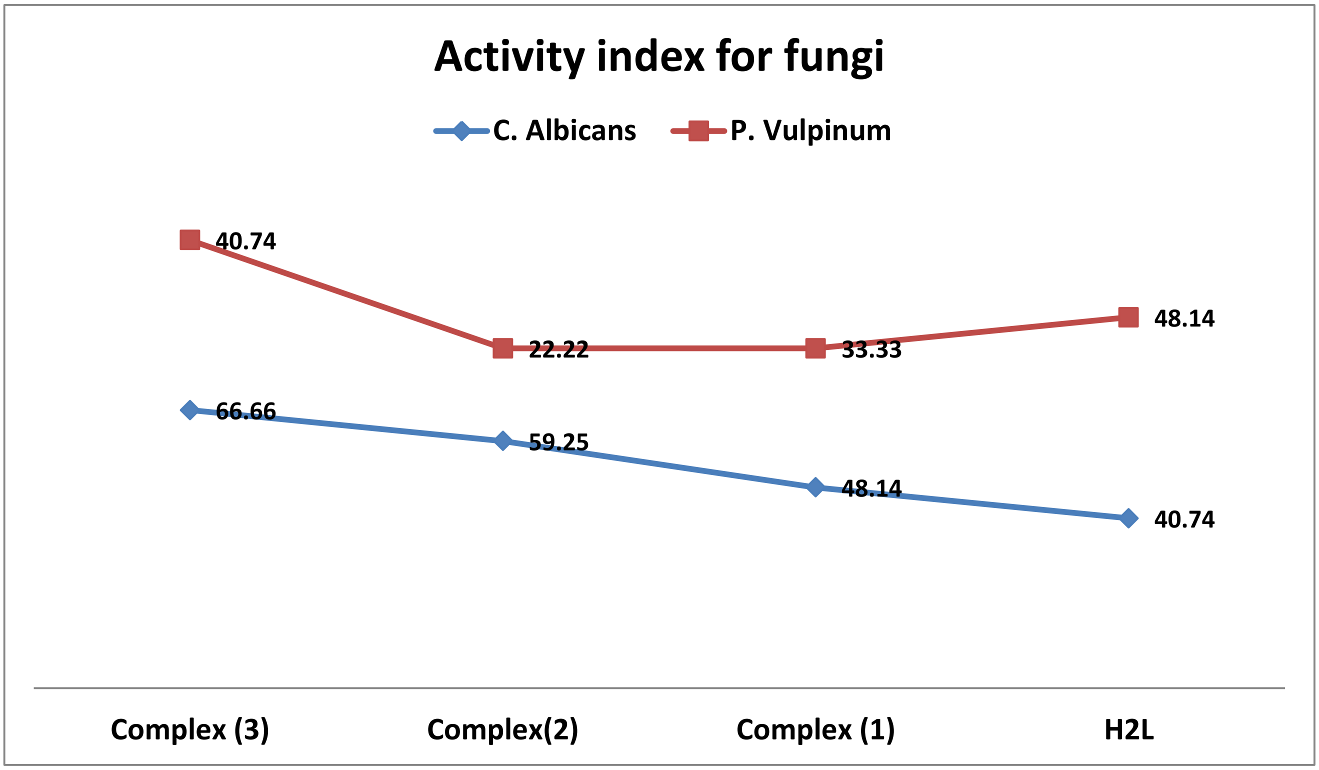

Figure S9 Activity index for fungi strains of **H_2L** and its metal complexes
